# Development of the Migrant Friendly Maternity Care Questionnaire (MFMCQ) for migrants to Western societies: an international Delphi consensus process

**DOI:** 10.1186/1471-2393-14-200

**Published:** 2014-06-10

**Authors:** Anita J Gagnon, Rebecca DeBruyn, Birgitta Essén, Mika Gissler, Maureen Heaman, Zeinab Jeambey, Dineke Korfker, Christine McCourt, Carolyn Roth, Jennifer Zeitlin, Rhonda Small

**Affiliations:** 1 Ingram School of Nursing and Department of Obstetrics and Gynaecology, McGill University, 3506 University St., Room 207, Montreal, Quebec H3A 2A7, Canada; 2 McGill University Health Centre Research Institute, Montreal, QC, Canada; 3 Ingram School of Nursing, McGill University, Montreal, Quebec, Canada; 4 Department of Women’s and Children’s Health, International Maternal and Child Health (IMCH), Uppsala University, Uppsala, Sweden; 5 THL National Institute for Health and Welfare, Helsinki, Finland and NHV Nordic School of Public Health, Gothenburg, Sweden; 6 Faculty of Nursing, University of Manitoba, Winnipeg, Manitoba, Canada; 7 Ingram School of Nursing, McGill University, Montreal, Quebec, Canada; 8 Netherlands Organization for Applied Scientific Research TNO, Department of Child Health, Leiden, Netherlands; 9 Midwifery and Child Health, School of Community and Health Sciences, City University, London, UK; 10 School of Nursing & Midwifery, Faculty of Health, Keele University, Staffordshire, UK; 11 INSERM, UMR S953 Epidemiological Research Unit on Perinatal Health and Women's and Children’s Health, Université Pierre et Marie Curie-Paris 6, Paris, France; 12 Judith Lumley Centre, La Trobe University, Melbourne, Victoria, Australia

**Keywords:** Childbirth, Ethnicity, Immigration and emigration, Maternal-child health services, Patient-centred care, Patient satisfaction, Pregnancy, Quality of health care, Questionnaires, Women

## Abstract

**Background:**

Through the World Health Assembly Resolution, ‘Health of Migrants’, the international community has identified migrant health as a priority. Recommendations for general hospital care for international migrants in receiving-countries have been put forward by the *Migrant Friendly Hospital Initiative*; adaptations of these recommendations specific to maternity care have yet to be elucidated and validated. We aimed to develop a questionnaire measuring migrant-friendly maternity care (MFMC) which could be used in a range of maternity care settings and countries.

**Methods:**

This study was conducted in four stages. First, questions related to migrant friendly maternity care were identified from existing questionnaires including the *Migrant Friendliness Quality Questionnaire*, developed in Europe to capture recommended general hospital care for migrants, and the *Mothers In a New Country (MINC) Questionnaire*, developed in Australia and revised for use in Canada to capture the maternity care experiences of migrant women, and combined to create an initial MFMC questionnaire. Second, a Delphi consensus process in three rounds with a panel of 89 experts in perinatal health and migration from 17 countries was undertaken to identify priority themes and questions as well as to clarify wording and format. Third, the draft questionnaire was translated from English to French and Spanish and back-translated and subsequently culturally validated (assessed for cultural appropriateness) by migrant women. Fourth, the questionnaire was piloted with migrant women who had recently given birth in Montreal, Canada.

**Results:**

A 112-item questionnaire on maternity care from pregnancy, through labour and birth, to postpartum care, and including items on maternal socio-demographic, migration and obstetrical characteristics, and perceptions of care, has been created - the *Migrant Friendly Maternity Care Questionnaire (MFMCQ)* – in three languages (English, French and Spanish). It is completed in 45 minutes via interview administration several months post-birth.

**Conclusions:**

A 4-stage process of questionnaire development with international experts in migrant reproductive health and research resulted in the MFMCQ, a questionnaire measuring key aspects of migrant-sensitive maternity care. The MFMCQ is available for further translation and use to examine and compare care and perceptions of care within and across countries, and by key socio-demographic, migration, and obstetrical characteristics of migrant women.

## Background

In 2010 there were an estimated 214 million migrants (individuals born outside the country in which they currently live) worldwide, half of whom were women [1]. Through the World Health Assembly Resolution, ‘Health of Migrants’, the international community identified migrant health as a priority, recognizing the health of migrants as a human right and calling for World Health Organization Member States to promote migrant-sensitive health policies and programs [2]. The *Report of a Global Consultation on the Health of Migrants – the Way Forward* summarizes a consultation convened in response to the Resolution in which several priorities were identified, including ensuring health systems are migrant-sensitive [3].

In 2004, the Migrant Friendly Hospitals (MFH) Project, a European initiative to promote migrant health and health literacy, published the *Amsterdam Declaration*, including recommendations for health professionals working in hospitals [4], based on an extensive review of the literature [5]. Twenty-six recommendations were made within six themes: development of services/organizational cultures; owners/management; staff/health professionals; users/representatives of the community; health policy/administration; and health science. From the 26 recommendations, a questionnaire was developed for completion by hospital managers in 12 hospitals in as many countries and was used as a self-evaluation tool before and after activities had been created in response to the recommendations [6]. While the MFH project offers recommendations for providing optimal care to hospitalized migrants generally and did carry out a sub-project on maternity care [7], its primary focus was not maternity care and women’s perspectives of care were not assessed.

Evidence suggests that the health of migrant women would benefit from specific consideration with regard to equitable maternity care. Reproductive Outcomes And Migration (ROAM), an international research collaboration, was established in 2005 to identify migrant reproductive health disparities, their causes, and approaches to reduce them. ROAM identified 133 reports with information on more than 20 million migrants in a systematic review of 13 years of literature [8]. Over half of all studies reviewed showed that migrants had worse outcomes compared with receiving-country-born women on fetal and infant mortality, caesarean birth, maternal health, congenital defects, prenatal care, and infection. Meta-analyses showed that Asian, North African, and sub-Saharan African migrants were at greater risk for fetal and infant mortality than ‘majority’ receiving populations. Conversely, in over half of all studies, migrants did the same or better than receiving-country-born women on preterm birth, low birth weight and health promoting behaviours. More focused ROAM reviews found that certain groups of migrant women were more likely to have preterm birth [9] and inadequate prenatal care [10] and that adjustment of background factors in several studies did not explain excess fetal and infant mortality risk in infants born to migrant women compared to those born to receiving-country women [11]. Another ROAM study found that infants of migrant Somali women experienced excess mortality and excess caesarean birth in spite of being less likely to be born preterm or to be of low birth weight [12]. Other studies, reporting on user-perceived measures such as satisfaction with maternity care and feeling treated with respect by health care providers, have also noted differences between migrant groups in some countries [13-15]. These studies highlight the need to go beyond conventional bio-medical risk factors to explore other possible mechanisms which could explain perinatal health differences between migrants and non-migrants; in fact, aspects of equity in health care delivery may have an important role in the generation of these disparities.

Equity in health is described as “the absence of systematic or potentially remedial differences in one or more aspects of health across populations or population groups defined socially, economically, demographically or geographically [16]”. There is mounting evidence that health care systems can mediate or contribute to inequalities in health, depending on health care access, utilization, and quality differences between population sub-groups [17-19]. We have conceptualized ‘migrant friendly maternity care’ (MFMC) as encompassing physical and psychosocial care by professionals with international migrants that are supportive in nature and specific to care provided during pregnancy, birth, or post-birth either in or outside hospital settings. One particularly important facet of migrant-sensitive care is communication. Barriers to communication have been shown to lead to adverse effects on: quality of care, user satisfaction, health outcomes, resource utilization for diagnostic testing, unnecessary invasive procedures, barriers to continuity of care, use of preventive services, and mortality [20,21]. Within maternity settings, the offer and use of culturally-trained interpreters, culturally/linguistically appropriate educational materials, and avoidance of the use of children as interpreters are valuable approaches to help migrant women navigate their way through the health care system. Furthermore, interventions fostering social support such as greater family visitation and use of community resources constitute important tools of MFMC.

To our knowledge, no available questionnaire measures MFMC for the purpose of cross national comparisons, and none seeks the views of women who are themselves using maternity services. Assessment of the extent to which this care is already being given, and the effectiveness of programs to optimize migrant-sensitive maternity care, is hampered by the absence of a measurement tool to do so. We therefore sought to develop a questionnaire measuring MFMC which could ultimately be applied in a range of maternity care settings and countries to permit local, national, and international comparisons to be made.

## Methods

A core project team of nine self-selected ROAM members was assembled to oversee the study. Representation was from a range of countries including Australia, Canada, Finland, France, the Netherlands, Sweden, and the UK. The study was conducted in four stages. First, questions related to MFMC were identified from existing questionnaires and combined to create a long-form questionnaire. Second, a Delphi consensus process with a panel of international experts was undertaken to identify priority themes and questions as well as wording and format (content validation). Third, the draft questionnaire was translated from English to French and Spanish and back-translated and was culturally validated (assessed for cultural appropriateness) by recent migrant mothers (face validation). Fourth, the questionnaire was piloted with migrant women who had recently given birth in Montreal, Canada.

### Stage 1. Review of existing questionnaires and identification of MFMC questions

The initial questionnaire was drafted from components of existing questionnaires including the *Migrant Friendliness Quality Questionnaire*, developed and used in twelve European hospitals to capture recommended general care for migrants [6], and the *Mothers In a New Country (MINC) Questionnaire*, developed and used in Australia [13,22,23] and revised for use in Canada by altering particulars of English language use that differed between the two countries, how incomes were reported, etc. [24]. We also adapted questions from the *Canadian Maternity Experiences Survey*[25] and other questionnaires developed and used previously by one or more authors [26,27]. The project team recognised that this initial questionnaire was incomplete and also required cross-national consultation and agreement; hence, identification of themes and related questions thought important to capture in a questionnaire on migrant-sensitive maternity care became the goal of Round 1 of the Delphi process.

### Stage 2. Delphi consensus process

A Delphi consensus process in three rounds was conducted. A Delphi process is the use of structured, individual questionnaires to elicit a group opinion from a panel of experts in a certain field [28]. In this case, we sought to elicit feedback on a questionnaire to measure MFMC from the perspective of migrant women who recently gave birth. An invitation to participate in the Delphi process was sent to all ROAM members and to others identified by ROAM members as having expertise in MFMC. A panel of 89 research and clinical experts in maternity care and migration from 17 countries was formed; each panel member responded to at least one of the three rounds.

The project team decided it would be important to gather data from the Delphi panel with regard to prioritized key topics for capture in such a questionnaire and to ensure that those themes and related questions were incorporated into the initial draft questionnaire. Hence, in Round 1, themes and questions to measure those themes were solicited. In Round 2, the usefulness of the proposed questions and response options was assessed and recommendations for how to reduce the length of the questionnaire were solicited. In Round 3, ‘core’ questions to be used in international comparisons were identified, questions were clarified or eliminated, and feedback on administration of the questionnaire was given. Questions were designated as ‘core’ if they fit the following criteria: ≥ 85% of respondents rated the question as “important” or “essential”; the question received a frequency rating of two or more in response to, “If you could ask only 10 questions from the questionnaire you have reviewed, which 10 would you ask?”; or if it was a new question developed in response to comments from Round 2 (unless it was directly associated with a question identified for elimination). At the completion of each round, quantitative data were analysed descriptively using means and frequency tables and textual data were summarized in tabular form. These results were then shared with the panel in the next round together with a new set of questions based on the results and on the goals of the next round.

### Stage 3. Cultural validation of the proposed questionnaire

At the completion of the Delphi process, the questionnaire was translated from English to French and Spanish, and back-translated to English, and all three language versions were revised through discussion by translators to ensure conceptual equivalence across the three languages. The questionnaire was subsequently culturally validated by migrant women discussing the questionnaire in groups using the questionnaire language of their choice. Nine women who had responded to advertisements and self-identified as being new to Canada within the last five years, giving birth within the last three to six months, speaking English, French, or Spanish and available for three hours during specified dates were commissioned to review the questionnaire for relevance and cultural appropriateness. Further adjustments to the questionnaire were made based on their feedback. Details of this process and related references can be found in Additional file 1.

### Stage 4. Piloting the questionnaire among migrant women in Montreal, Canada

The culturally-validated questionnaire was then pilot tested with migrant women post-birth. Thirty-three women from a number of different countries who gave birth on one of four maternity units in Montreal were recruited and consented (in writing or verbally) to participate between January-April 2011. Criteria for study participation by these women included being in Canada for 10 years or less, planning to remain in Montreal post-birth, and speaking English, French or Spanish. In addition, priority was given to women having refugee or asylum seeker status since previous work had shown these migrant women to have the greatest difficulty in having their pregnancy-related health concerns addressed [29]. Ethical approval for the study was obtained from the Genetics/Population research/Investigator initiated research (GEN) Research Ethics Board of the McGill University Health Centre. Women were administered the draft MFMCQ via telephone eight months post-birth. Questionnaire changes suggested through this process, by either the women themselves or the research assistants who administered the questionnaire, were discussed by the project team, and resulting revisions were translated and back-translated again across the three languages to maintain equivalency.

## Results

**Figure 1:**
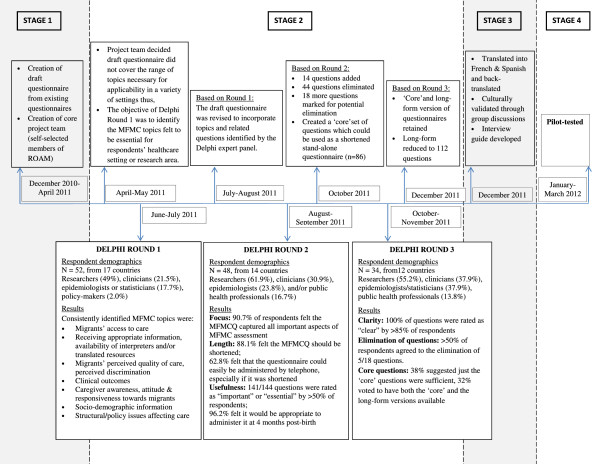
Development of the Migrant Friendly Maternity Care Questionnaire (MFMCQ).

The project was conducted over a 16-month period. The timeline of the study together with the Results are graphically depicted in Figure 1. The number of respondents in the Delphi panel was 52 in Round 1 (from 17 countries), 48 in Round 2 (from 14 countries) and 34 in Round 3 (from 12 countries). Panellists held multiple roles, with three-quarters self-identifying as perinatal researchers or epidemiologists and half as clinicians or public health professionals. In Round 1 of the Delphi, the themes highlighted by the panel for inclusion in a questionnaire on migrant-sensitive maternity care were:

•access to care;

•information exchange – verbal and written;

•migrants’ perceptions of care;

•clinical risks and outcomes;

•caregiver awareness, attitudes, and responsiveness towards migrants;

•socio-demographic characteristics of migrants; and

•structural issues affecting care.

The draft questionnaire reviewed in Round 2 incorporated the themes and related questions identified in Round 1. This more comprehensive version of the questionnaire was assessed by over 90% of panellists in Round 2 to have captured all key elements. In response to feasibility-related questions posed in Round 2, nearly 90% reported that the questionnaire should be shortened yet this was made difficult by the fact that over 50% reported that nearly every question (141/144) was “important” or “essential”. Two-thirds felt that the questionnaire could easily be administered by telephone, especially if it was shortened, and the vast majority felt it would be appropriate to administer it at four months post-birth.

During Round 3, in assessing the next draft of the questionnaire for clarity of each question and set of response options, and to identify a ‘core’ set of questions, 85% of the panel rated all questions as “clear”; and of the 18 questions suggested for elimination, only 5 were agreed to be eliminated by over 50% of panellists. Eighty-six questions were identified as a minimum set of questions for use in international comparisons (identified by * in the MFMCQs in the Additional files) and approximately one-third of the panel felt that inclusion of only those questions was sufficient as a questionnaire. An equal number felt that both the ‘core’ questions and other questions should comprise the final questionnaire; therefore both sets of questions were retained.

Women reviewing the questionnaire for cultural appropriateness provided feedback on how best to conduct the interview (e.g., once you know who the primary health care provider was for a given mother, keep referring to that specific health care provider during the interview). They also suggested more detailed information be given about the purpose of the questionnaire and of certain specific questions to help optimize overall response rates and minimize item non-response. These comments, and others, highlighted the need for a detailed interview guide, which was subsequently developed. Additional feedback was given on specific questions with suggestions for rewording to enhance clarification or universality, and this feedback was reviewed by the project team and used to revise the questionnaire further.

Feasibility and ease of administration was assessed during pilot testing. The average duration of the interviews was 45 minutes. Interviewers made recommendations for minor revisions to the questionnaire to improve clarity of the questions. The project team examined the few items with high non-response rates to assess whether re-wording or revising the order of the questions might decrease these rates; this resulted in minor changes. The final 112-item MFMCQ and Guide for Interviewers in English, French, and Spanish can be found in Additional files 2, 3, and 4, respectively.

Table 1 shows the data captured by the MFMCQ questions by variable groupings (i.e., migration, health care services, current and past obstetrical history, migrant women’s perceptions of care, and socio-demographic characteristics) and showing coverage of the key themes previously identified in the Delphi process as important [access to care (ATC), information exchange (IE), perceptions of care (PC), clinical risks and outcomes (CRO), caregiver awareness and responsiveness (CAR), socio-demographic characteristics (SDC), and structural issues (SI)]. In this way, a completed MFMCQ offers both descriptive data on MFMC provided, which could be used as baseline data for program planning and evaluation, but also offers the possibility of examining associations between key socio-demographic, migration, and obstetrical indicators and MFMC within the population of respondents at a single point in time.

**Table 1:** Data captured by the MFMCQ

**Variable**	**Key theme measured**^**1**^	**MFMCQ question number**
***Migration***		
Country of birth	MR	1
Length of time in receiving country	MR	2
Arrived in receiving country pregnant	MR	3
Number of children born in receiving country	MR	88
Countries of birth of mother’s parents	MR	90, 91
Immigration status	MR	92, 93, 94, 95, 96
Spent time in detention centre	MR	97, 98, 99
Permitted to work in receiving country	MR	102
Language spoken at home	MR	108
Fluency in receiving-country language(s)	MR; IE	109, 110
***Health care services***		
Cared for by health care professional (HCP)	ATC; SI	4, 5
Prenatal care	ATC; SI	6, 7
Services during pregnancy	ATC; SI	9
Given information in language known to migrant	IE	13
HCP asked how planned to infant feed	ATC; CAR	15
HCP asked if preferences for care	ATC; CAR	16
Site of birth	ATC; SI	20
Type of HCP during labour, birth	ATC; SI	21, 22
Procedures during labour, birth (e.g., caesarean)	ATC; CAR; SI	23
Allowed to move around, choose positions during labour	ATC; CAR	26
HCP asked re: preferences for pain management during labour	ATC; CAR	27
During labour, allowed to have choice of support people	ATC; CAR	29
During birth, had a companion	ATC	30, 31
HCP asked re: preferences for care during labour, after birth	ATC; CAR	32, 37
Infant admitted to special care unit	CRO	33
Length of hospital stay	ATC; SI	34
HCP asked re: food preferences	ATC; CAR	36
Given baby to hold skin-to-skin within first hour after birth	ATC	38
HCP offered help/info re: breastfeeding	ATC; CAR	39, 40
BF support services used	ATC	41
HCP seen since birth	ATC; SI	42, 43, 44
HCP offered interpreting service	IE; ATC; SI	57
Frequency of interpreter in attendance and who acted as interpreter	IE; SI	58, 59
HCP asked if any questions	ATC; CAR	67
HCP kept woman informed	IE	71
***Obstetrics –current pregnancy***		
Medical complications pregnancy, labour, birth	CRO	8, 24
Gestational age at birth	CRO	17
Number of infants born	CRO	18
Infant birth weight	CRO	19
If caesarean birth, reason	CRO	25
***Obstetrics –history***		
Pregnancies (i.e.., gradivity)	CRO	78
Miscarriages	CRO	79
Terminations	CRO	80
Stillbirths	CRO	81
Infants born before 37 completed weeks	CRO	82
Infants born after 37 weeks	CRO	83
Medical complications during previous pregnancies	CRO	84, 85
***Perceptions of care***		
Services wished for but not used	PC	10, 11
Sources of information	PC	12
Received enough information	PC	14
Satisfied with how HCP helped to manage pain during labour	PC	28
Time in hospital/clinic post birth was adequate	PC	35
Wanted to see a health care professional but could not	PC	45, 46
Other advice/support/information wished for	PC	47
Felt welcomed by HCP	PC	48
Felt respected by HCP	PC	49
HCP were helpful	PC	50
Happy with care received	PC	51
Was asked by HCP to do something woman did not want to do	PC	52, 53
HCP asked preferences for female/male HCP	PC	54
Understood info provided by HCP	PC	55
Would have understood info better in another language	PC	56
Satisfaction with interpretation	PC	60
Had preferences for care but they couldn’t be followed	PC	61, 62, 63
Things HCP could do differently/better	PC	64, 65
Particularly good/bad experiences	PC	66
HCPs were rushed	PC	68
Concerns taken seriously by HCPs	PC	69
Wait too long for care	PC	70
Felt comfortable asking about things not understood	PC	72
HCPs made decisions without women’s wishes being taken into account	PC	73
HCPs encouraging and reassuring	PC	74
HCPs spent enough time providing explanations	PC	75
Thought to be treated differently to other people by HCPs	PC	76, 77
***Socio-demographics***		
Marital status	SDC	86
Household composition	SDC	87, 88
Maternal birth date	SDC	90
Health services funding	SDC	101
Education	SDC	102
Employment	SDC	104, 105, 106
Household income	SDC	107, 108

^**1**^ATC = Access to care (n ≥ 21 questions).

CAR = Caregiver awareness and responsiveness (n ≥ 10 questions).

CRO = Clinical risks and outcomes (n ≥ 13 questions).

IE = Information exchange (n ≥ 5 questions).

MR = Migration-related (n ≥ 10 questions).

PC = Perceptions of care (n ≥ 27 questions).

SDC = Socio-demographic characteristics (n ≥ 7 questions).

SI = Structural issues (n ≥ 10 questions).

## Discussion

We have developed the MFMCQ, a questionnaire measuring migrant-sensitive maternity care, for use in a range of maternity care settings and countries. The MFMCQ is currently available in three languages and can be used in at least two ways. First, it can be used as a stand-alone questionnaire to assess migrant friendly maternity care within a country or setting or across countries or settings (our primary purpose in developing it). Second, it can be used as a bank of questions that address challenges women might face as migrants. In this second scenario, specific questions could be selected for use to complement other approaches to data collection. For example, all MFMCQ questions may be retained except for those on obstetrical history if medical record reviews are being conducted to gather these latter data. Also under this second scenario, MFMCQ questions could be selected based on narrower research questions. For example, if one project is meant to focus exclusively on the birth event, investigators could choose to use only those questions or portion of questions pertaining to birth and not use those related to maternity care pre- and post-birth. We encourage users of the MFMCQ to contact us to share their experiences with the questionnaire with a view towards optimizing its use in a range of countries and settings. See the *Translation and Cultural Validation Protocol* (Additional file 1) for details of the recommended process.

Development of the MFMCQ responds to the World Health Assembly Resolution, ‘Health of Migrants’, by offering a means by which migrant-sensitive care (in this case, related to pregnancy and birth) can be monitored as one of the specific priority areas identified in the *Report of a Global Consultation on the Health of Migrants – the Way Forward*[3]. Monitoring was described as ensuring the standardization and comparability of data on migrant health; supporting the appropriate aggregation and assembling of migrant health information; and mapping good practices in monitoring migrant health, policy models, and health system models. The MFMCQ was developed by an international panel of experts with a view towards permitting data specific to migrant perinatal health and care to be collected and compared across settings and countries.

Extensive worldwide movement of individuals and evidence that health disparities exist between migrants and receiving-country nationals has stimulated interest in the issue of health equity for migrants. Differences in perinatal health outcomes and care have been documented in many countries [13,15,30,31]. The direction of these differences varies as was seen in systematic reviews of the literature by ROAM members [8-11]. In some countries, migrant women and their infants have worse perinatal health outcomes [15,30,32,33] while in others, migrant women have outcomes comparable to or better than those of host-country women [31,34,35]. These results suggest that social, medical, and health system factors have an important mediating role in the generation of health disparities. The delivery of health care services can be inequitable if there are barriers (such as communication problems, or prejudicial attitudes) to some groups receiving care from health professionals or if services are delivered in a way deemed unsatisfactory to the population they are intended to serve.

International comparative studies are needed to generate the evidence necessary to consider the full array of mechanisms by which migrant women experience better or worse health outcomes in some countries than others. This requires research strategies that can be applied across a range of countries with different health care and immigration systems [36]. Whitehead (1992) argues that it is only by monitoring the acceptability of care provision with service users that equity of service delivery can truly be measured [37]. Development of methods to measure migrant-sensitive care from the perspectives of international migrants themselves has been limited, likely due to the challenges inherent in creating instruments which are multilingual and culturally appropriate for use with different migrant populations. The MFMCQ is the only tool of which we are aware that can be used for this purpose with respect to assessing equitable maternity care for migrants.

In order to compare studies of migrants internationally, common indicators of migration must be agreed upon and routinely collected. A previous Delphi process involving a panel of international experts reached consensus on how to record indicators of cross-border migration as they relate to perinatal health [38]. For routine and population-based perinatal health surveillance, ‘country of birth’ and ‘length of time in receiving-country’ were recommended. The indicators ‘immigration status’, ‘receiving-country language fluency’, and ‘ethnicity’ (as defined by maternal parents’ place of birth), were suggested for specific research studies or surveillance modules to complement routine data collection [38]. These five migration indicators have been incorporated into the MFMCQ.

Comparing the care received by migrant women and their health outcomes in various countries can contribute to the identification of factors associated with optimal care such that health policy makers and managers might improve services by sensitively adapting approaches that have worked in other countries. International research collaborations can enhance knowledge-creation and translation within and across countries by pooling research capacities, exploiting ‘natural experiments’ in policy and practice, and replicating effective population interventions [39]. Additional translation and piloting of the questionnaire in other languages and countries is required and is being planned. This will expand the number of countries in which comparisons will ultimately be feasible. Approaches to translation have been summarised elsewhere [40,41] and our approach is similar to those generally recommended. Although the length of the questionnaire did not pose difficulties during our pilot, future identification of a sub-set of questions, which predicts findings from the larger set, may be warranted.

Our process to create a common questionnaire was not without limitations. We did not assess test-retest reliability, which is important to assure as a fundamental part of psychometric testing (although test-rest reliability in a pregnancy-birth context is quite challenging since views of care received often change considerably as time since birth lengthens). In addition, the first use of the questionnaire was limited to a single country and three languages. Differences in health care delivery systems may result in a need to introduce slight modifications to the questionnaire to suit local circumstances. The same is true for socio-demographic background factors. With translation of the questionnaire into further languages, additional modifications or simplification may also be required in order to maximise comparability between the translated versions. It is our hope that as the questionnaire is used in different settings and countries and it is translated into different languages, experiences of its use will be reported and shared, enabling any needed improvements to be incorporated. In addition, although all elements defined by the expert panel as key to be measured in a questionnaire of this type were included in the MFMCQ, some elements were covered to a greater extent than others. For example, although structural issues are captured in the MFMCQ, it may be appropriate to supplement data obtained from women with data gathered by other means such as questionnaires administered to managers (as in the original MFHI) or through record review or direct observations of care. Application of the US National Culturally and Linguistically Appropriate Services (CLAS) Standards in Health and Health Care [42] and MFH [4] standards to assess structural issues in care provision are also required for hospitals to assess how they are performing and to improve care.

## Conclusions

A four-stage project including a three-round consensus process of questionnaire development with international experts in migrant reproductive health and research resulted in the MFMCQ, a questionnaire assessing key aspects of migrant-sensitive maternity care. The MFMCQ is available for use to examine and compare care and perceptions of care within and across countries, and by key socio-demographic, migration, and obstetrical characteristics of migrant women who have recently given birth.

## Competing interests

The authors declare that they have no competing interests.

## Authors’ contributions

AG conceived of the study, participated in its design, obtained funding, had responsibility of general study oversight, contributed to the analyses, and drafted the manuscript. ZJ coordinated the hospital data collection and recruitment which enabled the piloting of the MFMCQ, translated the English version of the MFMCQ into Spanish and obtained English back-translations for that version, and pilot-tested the Spanish MFMCQ. RD coordinated the Delphi process and revised the questionnaire as needed at each phase. The remaining authors: BE, MG, MH, DK, CM, CR, RS, and JZ provided input on the content, format, and length of the questionnaire as well as wording of specific questions. They also suggested potential participants for the Delphi process. All authors contributed to analyses of the results and to the intellectual content of the paper and approved the final version to be published.

## Authors’ information

AG is co-leader of the international research collaboration, ROAM (Reproductive Outcomes And Migration), and coordinator of the Global Health Studies section of the Master’s program in nursing at McGill University. RD is a recent graduate of the Global Health Studies section of the Master’s program in nursing at McGill University. BE is a member of ROAM, a Senior Lecturer in International Maternal and Reproductive Health at Uppsala University, and a Consultant Obstetrician at Uppsala University Hospital, Sweden. MG is a steering committee member of the international research collaboration, ROAM, and Research Professor at the Information Department at the National Institute for Health and Welfare (THL) in Helsinki, Finland and a Professor at the Nordic School of Public Health, Gothenburg, Sweden. MH is a Professor and Canadian Institutes of Health Research (CIHR) Chair in Gender and Health, Faculty of Nursing, University of Manitoba, and member of ROAM. ZJ is completing graduate studies in ‘Innovation in tourism management, specializing in gastronomic and culinary heritage management’ at the University of Barcelona. DK is member of the international research collaboration, ROAM, researcher at TNO (Netherlands) in Medical Anthropological and Reproductive Health and Executive Secretary of the Preparing for Life Initiative. RS is co-leader of the international research collaboration, ROAM, and Professor at the Judith Lumley Centre at La Trobe University in Australia.

## Pre-publication history

The pre-publication history for this paper can be accessed here:

http://www.biomedcentral.com/1471-2393/14/200/prepub

## Supplementary Material

Additional file 1 MFMCQ Trans&Val Protocol_17Apr2014.pdf: the MFMCQ Translation and Cultural Validation Protocol. Click here for file

Additional file 2 MFMCQ English version_17Apr2014.pdf: the MFMCQ in English. Click here for file

Additional file 3 MFMCQ French version_17Apr2014.pdf: the MFMCQ in French. Click here for file

Additional file 4 MFMCQ Spanish version_17Apr2014.pdf: the MFMCQ in Spanish. Click here for file
