# Supplementary material for: Development of the Migrant Friendly Maternity Care Questionnaire (MFMCQ) for migrants to Western societies: an international Delphi consensus process
Source: BMC Pregnancy Childbirth. 2014 Jun 10;14:200. doi: 10.1186/1471-2393-14-200 (PMC4088918; doi:10.1186/1471-2393-14-200)
Supplement: Additional file 1 — MFMCQ Trans&Val Protocol_17Apr2014.pdf: the MFMCQ Translation and Cultural Validation Protocol. [file 1471-2393-14-200-S1.pdf]

MFMCQ

(Migrant Friendly Maternity Care Questionnaire)

Translation and Cultural Validation Protocol

Reproductive Outcomes and Migration (ROAM) International Research Collaboration

April 17, 2014

Table of Contents

DEFINITIONS **3**

TRANSLATION & CULTURAL VALIDATION STEPS **4**

1. Creation of and consultation with an advisory committee 4
2. Translation & adaptation of the questionnaire 4
3. Assessment of the readability of the questionnaire 5
4. Blind back-translation of the questionnaire 5
5. Discussion with Ethno-Cultural Liaison Groups (ECLGs) 6
6. Incorporation of ECLG results 10
7. Administration of translated questionnaires to monolingual women 11
8. Pilot-testing of the final target language version questionnaire 12
9. Provision of the final target language questionnaire to ROAM 12

MFMCQ TRANSLATION & CULTURAL VALIDATION COMPANION CHECKLIST **13**

REFERENCES **18**

Definitions

Adaptation: the changes required in addition to simple translation to ensure the content, cultural references, and wording of the questionnaire are appropriate to the intended respondents[1]

Advisory committee: a group of researchers, health care professionals, and representatives from non-government organizations and government agencies interested in migration and the reproductive health of migrant women and willing to inform research in this area

Bilingual: the ability to speak the source language of one of the culturally validated MFMCQs and the new target language fluently

Bilingual liaisons: individuals from the target language communities who have written and spoken proficiency and cross-cultural competence in both the culturally validated MFMCQ language and at least one target language; they serve as mediators between groups or persons of different cultural backgrounds[2]

Ethno-cultural liaison group (ECLG): a group of women who reflect the target population and its ethno-cultural communities and languages

Fluent: the ability to speak and write easily in the language of interest, and familiarity with the culture associated with the language

Idiom: a term whose meaning cannot be deduced from its individual words (i.e., its figurative meaning differs from its literal meaning)

Monolingual: speaks only the target language (although may speak dialects of the target region)

Native language: the language that a person has spoken from early childhood

Readability: a measure of how easy it is to read the questionnaire

Researcher: member of the research team

Source language: language of one of the culturally validated MFMCQs

Target language: language of the MFMCQ to be validated

Translation: the process of transforming text from one language to another while ensuring conceptual equivalence

Translator: a bilingual individual who transforms an expression of a source language into an expression with a comparable meaning in a target language in written form[2]

Translation & Cultural Validation Steps

(Note: A related checklist follows)

1. Creation of and consultation with an advisory committee

Create a committee of researchers, health care professionals, and representatives from non-government organizations and government agencies interested in migration and reproductive health to advise the research team on the questionnaire content and formulation of questions, translation and cultural validation process.

2. Translation & adaptation of the questionnaire

Translate and adapt[1,3] the questionnaire from the source language (i.e., that of an already culturally validated MFMCQ language) to study population languages (i.e., target language). The intention is to have the questionnaire understood by a broad group of individuals who speak a given language, thus, translators with different backgrounds for each language should be hired as much as possible[4]. Select translators whose native language is the target language, who are fluent in both the source and the target language, and who are "intimately familiar" with the associated cultures.[3] Experienced translators are preferred.[3] Ideally, a translator will have learned the source and the target languages at different times and within different cultures for a richer and more accurate understanding of each culture.[5] Collect the following information for all translators: a) country of birth; b) length of time in this country; c) culture/ethnicity/religion; d) native language and where it was learned; e) other languages and where they were learned; f) demographic information (i.e., age and gender; g) education level.

Translators should consult the following guidelines adapted from Brislin's recommendations (1986)[6,7] for clear wording of research instruments:

1. Use short, simple sentences (i.e., 16 words or less)
2. Use active rather than the passive voice
3. Repeat nouns rather than using pronouns
4. Avoid metaphors and colloquialisms
5. Avoid the subjunctive (e.g. 'could', 'would', 'should', etc.)
6. Add sentences to provide context for key ideas
7. Avoid adverbs and prepositions telling 'where' or 'when' (e.g. 'frequently', 'beyond', 'upper', etc.)
8. Avoid possessive forms
9. Use specific rather than general terms
10. Avoid words indicating vagueness (e.g. 'probably', 'maybe', 'perhaps')
11. Use wording familiar to the translators
12. Avoid sentences with two different verbs if the verbs suggest two different actions

Certain words may be directly taken from the source language version if they are more commonly used or recognized than a target language translation might be (i.e., certain medical procedures, official immigration terms). These should be included in brackets or quotes in the target language version.[4]

The footer of each page of the new target language version of the MFMCQ should include "*MFMCQ – original translation to (*insert: *target language) – (*insert: *date)*." The date is key to ensure the most recent version is always used.

3. Assessment of the readability of the questionnaire

Assess how easy it is to read the translated questionnaire (readability) referring to the guidelines adapted from Brislin's recommendations listed in *Step 2: Translation and adaptation of the questionnaire*. Although developed to improve the translatability of English source languages, several points are applicable across all target language versions. For example, use short and simple sentences, and avoid colloquialisms.

If available, a readability tool may also be used. In English, the Simple Measure of Gobbledygook (SMOG) formula has been recommended for use in health literature[8], and the Flesch-Kincaid (F-K) readability formula, which is available in Microsoft Office® word processing software, has been found to be highly related to the scores generated using other formulae.[9] Scores are generated using numbers of syllables per word and number of words per sentence to produce estimates of the reading level required to understand a particular text. It is recommended that health literature be written at a level requiring no more than 5 years of formal education.[9]

Caution should be used in applying readability tests developed for the English language to other languages; findings of a study assessing conversion of SMOG scores between English, Spanish and French suggest potential systematic bias towards English being more easily readable.[10] Readability tools adapted specifically to the target language should be used. Note that readability tools are limited because they do not take into account factors influencing questionnaire comprehension such as content, cultural appropriateness, previous experience, interest, or the effect of new material.[9]

4. Blind back-translation of the questionnaire

Translate the target-language questionnaire back to the source language. Identify a translator who has not read the source language version of the questionnaire, and (if possible) who has a different background (culture, educational level, religion, etc.) than the original translator do the reverse ("back") translation. The back-translator should be fluent in both the source and target languages, and ideally should be a native speaker of the source language. If the back-translation is written, the back-translator should complete the back-translation independently and should not consult with the original translator.[3]

Alternatively, an oral back-translation process can be completed. In this method, the back-translator reads through the target language version and orally translates as he/she is reading. The researcher and the original translator are present, and discrepancies between the back-translator's understanding and the intended meaning are discussed immediately until both translators agree upon an acceptable wording for the translation. The back-translator should not have seen the source version although he/she may have been given the target language version to review prior to the meeting. This oral method may be particularly useful in cases where a language has only been written for a short period of time and hence, a great deal of discrepancy between the two translators might be expected.

Systematically compare the back-translated questionnaire with the source language version noting all discrepancies (differences between the intended meaning/essence of each question and what the back-translator understood). The researcher must discuss each discrepancy with both translators until agreement is reached on the optimal wording for the translated versions. The translators should also be asked to identify when multiple connotations of specific words are possible, and discuss whether words with more narrow interpretations can be substituted.[3]

When clarity is lacking in the source language version, researchers and translators should discuss how that version can be adjusted to make it clearer (e.g., remove ambiguous wording). Any suggested changes should be noted using track changes, "comments", or handwritten on a hard copy of the source language version. When time and resources permit, using more than two translators and repeating the back-translation process several times

improves results.[3,5] Any adjustment to the source version requires review for potential revision of all other existing language versions.

All changes made to the target version between translation and back-translation should be highlighted. The footer of each page of the new target language version of the MFMCQ after back-translation should include "*MFMCQ-post back-translation of (*insert: *target language) – (*insert: *date)*". The date ensures that the most up-to-date version is always used.

5. Discussion with Ethno-Cultural Liaison Groups (ECLGs)

Women who are proficient in speaking and writing at least one target language and the source language, have flexible availability, and are comfortable with the subject matter may be hired as bilingual liaisons. These bilingual liaisons will be responsible for recruiting ECLG members and monolingual women (for *Step 7*: *Administration of translated questionnaire to monolingual women*), and for acting as cultural brokers between the research team and the communities of which they are affiliated.

ECLG members (bi- or multilingual migrant women[11] reflective of a mix of target ethno-cultural communities and languages) can be recruited directly, through referrals from organizations working with these populations (e.g., the advisory committee), or using community advertising. Advertising should describe the type of ECLG member needed, the remuneration offered, and how to contact the research staff. These individuals are not considered research "subjects", rather as staff since they are informing the team on operational aspects of the study, not supplying data.

The profile of target ECLG members should reflect the study population as far as possible. They should:

- speak one of the study languages;
- have lived in the host country for ≤5 years;
- have given birth 3 to 6 months ago (or up to 1 year if not possible to find recently delivered women);
- be available in person for a 3 hour period and for 1 hour by phone 1-2 days prior to meeting;
- reflect a range of countries, ages, educational backgrounds, religions, length of time in host country, etc.

The 'Best fit' form (below) should be completed for each ECLG member.

Ethno-Cultural Liaison Group (ECLG)
'Best fit' form

Telephone questionnaire administration	
Completed ◻ ____________________ (dd/mm/yyyy)	Name: ________
Target language: ________________________	Sex: ◻ Female
ECLG availability:	Country of birth /origin: ________
	Sub-region of country: (e.g., north, south) ________
	Religion/Culture: ________
	Migration status: ◻ Naturalized Citizen<br>◻ Landed Immigrant<br>◻ Refugee<br>◻ Asylum seeker<br>◻ International Student<br>◻ Other: ______________

How long ago did you arrive in this country?	________
How old were you at the time?	________
How long ago did you give birth?	________
Did you arrive in this country pregnant?	◻ Yes ◻ No
How many years of schooling have you completed?	________
If currently employed what is your job?	________
If not currently employed what was your last job?	________
How many languages do you speak?	________
Mother tongue:	________

Language	Spoken	Read	Written
Source language: ________________________	◻Fluently<br>◻ Well<br>◻ With difficulty<br>◻ Not at all	◻Fluently<br>◻ Well<br>◻ With difficulty<br>◻ Not at all	◻Fluently<br>◻ Well<br>◻ With difficulty<br>◻ Not at all
Target language: ______________________	◻Fluently<br>◻ Well<br>◻ With difficulty<br>◻ Not at all	◻Fluently<br>◻ Well<br>◻ With difficulty<br>◻ Not at all	◻Fluently<br>◻ Well<br>◻ With difficulty<br>◻ Not at all
Other: ________________________	◻Fluently<br>◻ Well<br>◻ With difficulty<br>◻ Not at all	◻Fluently<br>◻ Well<br>◻ With difficulty<br>◻ Not at all	◻Fluently<br>◻ Well<br>◻ With difficulty<br>◻ Not at all

Are you an active member within your ethnic community?	◻ Yes<br>◻ Not really
Are you interested in being a representative of your ethnic community?	◻ Yes<br>◻ Not really
What knowledge or experience do you have with health and migration issues within this country?	
Do you feel you have contact with a wide variety of people from your community?	
Why do you feel you represent the views of your ethnic community?	

Potentials monolingual/bilingual Connects:

Name	Language	Contact #	Bi/mono
____________________________	________________________	________________	_________

Discussion groups should consist of 2-4 members fluent in the target language. Note: the presence and participation of bilingual liaisons can promote the discussions and explanations necessary for an effective ECLG meeting even in groups where the number of members is low. Diversity (i.e., countries of origin, ages, educational backgrounds, religions) within such groups increases the amount of explanation that members provide about meanings and understandings that may be implicit within their own cultural community.[11] The number of members consulted for the language being considered will depend on the number of variations and diversity of groups who speak the language (e.g., the Arabic of Lebanon and of Egypt are understood to be very different). Taken together, 3-4 members per language should be consulted – more or less depending on how broadly the language is spoken. This number is adequate when the aim is to assess clarity, wording, and acceptability of the study tool.[12]

One to two days before the group discussion, bilingual liaisons are to contact the women they identified for the ECLG to administer the questionnaire to them over the telephone. The bilingual liaisons should note any administrative issues that arise and the length of time it took to complete administration. This telephone call will also serve as reminder to the group members of the meeting. Members will be asked to share perceptions of their experiences responding to the questionnaire over the telephone at the group discussion.

Ask the members to assess each question and the questionnaire as a whole for:

a) cultural appropriateness and acceptability, and
b) feasibility of completion.

Cultural appropriateness can be assessed using a simple qualitative ranking tool (Figure 1) that allows members to assign an appropriateness score to the item in question.

Figure 1 Cultural Appropriateness Scale[11]

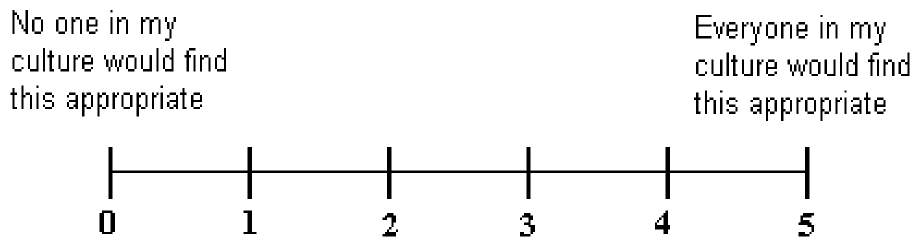

The cultural appropriateness scale[11] can be used as guide to prioritize the sections that require further group discussion. Sections scoring 0, 1 or 2 should be explored to determine if the acceptability of the pertinent questions can be improved.

Review any discrepancies between the groups' understanding of questions and the intended meaning of the questions. Asking members to paraphrase the meaning of a question or response option may sometimes be helpful. Provide babysitting services, a meal, and compensation to all members. Hot, sit-down meals are preferable to cold finger-food as they provide a more culturally-appropriate and welcoming setting for group interaction.[11] Detailed instructions on the process of conducting ECLG meetings are given in the text box below.

Instructions for conducting ECLG meetings

1. Read the following introduction to the ELCG members:

> *Thank you for agreeing to meet with us. We are working on a project called Migrant Friendly Maternity Care (MFMC). We want to know about care received by international migrant women during pregnancy, labour, birth, and after birth. Our goal for meeting with you is for us to learn from you whether a questionnaire we have developed about this care makes sense to you and whether there are ways you might suggest that we should improve it. An extensive process to create this questionnaire has already been completed with experts from 17 countries and it has been used in three languages.*
>
> *We plan on asking the questions in this questionnaire over the telephone to women about 4 months after birth. Because we will be asking questions over the telephone, we may have already called you and administered the questionnaire. If not, we would like to ask you the questions on the questionnaire verbally now and we will record your answers. We will not be keeping your answers. You are not required to give any information about yourself that you do not feel comfortable giving. The purpose of asking you the questions is only to have you think about what it will be like for other women who will be asked to complete this questionnaire and to help us determine how long it will take to complete it. We felt this was the best way for you to give us feedback on how the questionnaire might be improved. After you have completed the questionnaire, we would like to discuss each question and also discuss the questionnaire as a whole.*
>
> *You may be interested to know that this process is being done in several languages and in several countries. We will have some restrictions to what we can change in the questionnaire for scientific and/or resource reasons.*
>
> *Do you have any questions?*

2. If ECLG members have not yet answered the questionnaire, administer the target version MFMCQ to mother(s) (simulating a telephone call). Again, let the women know that we will not use their answers for anything, but we appreciate them answering as though they are study participants.

3. Time questionnaire completion.

4. Make notes as to which questions require clarification or that women are hesitant to answer. Also note any spelling errors, formatting issues, or other structural problems with the questionnaire that are brought out by the ECLGs.

5. Look for inconsistencies in answers and discuss hesitations.

6. Review questions found to be problematic. Ask women for their feedback on how to improve them.

7. Ask the following questions about the questionnaire as a whole:

i. What was your overall impression of the questionnaire?
ii. Have we missed any key topics from this questionnaire?
iii. Were there any questions that were uncomfortable for you to answer?
If yes, how can we change the wording?
iv. Were there any questions that were too personal?
If yes, how can we change the wording?

v. Were there any questions that were difficult to understand?
If yes, how can we change the wording?

vi. Were there any questions that were unclear?
If yes, how can we change the wording?

vii. Were there any questions that were not relevant?
If yes, how can we change the wording?

iii. Were there any questions that were harsh/too direct?
If yes, how can we change the wording?

ix. Are the questions appropriate for your culture?
(You may chose to use the cultural appropriateness scale)

x. Is there anything else that you feel would prevent other women with your cultural background from answering these questions?

xi. Does it flow well? Yes/No
If no, how would you change the order?

xii. What would make it easier to answer this questionnaire by telephone?
If the suggestion is to cut, then which questions?
If it is to divide telephone calls, then how many calls?

iii. Do you feel that responding to these questions at 4 months after birth will be difficult?
If yes, why?

iv. Do you have any suggestions about how to make responding to these questions easier?

8. Generate a list of names, contact information, and languages of potential women for monolingual testing (for Step 7) from the ECLG members.

9. Ask the members if they would be willing to be contacted by the research team again in regards to this or another project.

10. Pay the women for their time and obtain receipts.

6. Incorporation of ECLG results

All feedback from the ECLGs held for all languages should be summarized, and at least two researchers should systematically review the feedback item by item. Incorporate results for each language version from ECLG discussions into the questionnaire, recording all changes that are made and providing a brief rationale for each.

Modifications to the MFMCQ should be made if the issue:

- is raised by several discussion group members;
- is expected to compromise study participants' comfort and ability to respond.

Any suggested changes to the source language version should be noted using track changes, "comments", or handwritten on a hard copy of the source language version. Refer these suggested changes back to ROAM. You may wish to circulate these records to the advisory committee to keep them informed of recommended changes and for feedback as appropriate.

All changes made to the target version between back-translation and the ECLGs should be highlighted. The footer of each page of the new target language version of the MFMCQ after ECLG input should include "*MFMCQ- post ECLG of (*insert: *target language) – (*insert: *date)*". The date ensures that the most recent version is always used.

The table below is an example of a similar process used in another study for the question: "What is your ethnicity?"

Example of the translation and cultural validation process applied to a question in another study[4]					
**Original Question**	**Input from key players**	**Input from blind back-translation**	**Input from ECLGs**	**Input through non-response rates from pilot administration & additional feedback from key players**	**Final Question**
What is your ethnicity?	-Originally the question was grouped with migration questions. It was subsequently grouped with general information questions.<br>-Response options were suggested to facilitate answering the question and were added:<br>• African<br>• African-Canadian (American)<br>• Asian & Pacific<br>• Asian-Canadian (American)<br>• Eastern-European<br>• Western-European<br>• Scandinavian<br>• European-Canadian (American)<br>• Jewish<br>• Latin-American<br>• Hispanic<br>• Middle Eastern<br>• Native Canadian (American)<br>• Canadian<br>• Other:______	-Suggested removing "(American)"<br>-Suggested adding "Caribbean" as an option<br>-Term "ethnicity" wasn't understood and came back in the back-translation as: race, nationality, ethnic belonging, ethnic origin<br>-Generally there was difficulty across all languages to translate these terms:<br>e.g. Native Canadian was understood as "born in Canada" rather than as referring to "Aboriginal people"<br>-Some terms didn't exist in certain languages<br>e.g. there is no word for "Hispanic" in Urdu	"I am not sure how to interpret this. I am not sure that I would put myself in the responses"<br>-Women didn't understand what was really meant by "ethnicity" & were confused by the response options because they felt they represented geographical areas rather than ethnicity<br>-They had difficulty answering the question themselves.	-Before the questionnaire was used in the study the response options were removed & the question was left open-ended<br>-Women didn't understand the question & many women left it blank	-The question was removed

7. Administration of translated questionnaires to monolingual women

This step is most important when the proportion of the target population having no host country language capability is expected to be high (e.g., very newly arrived refugees). If needed and if time and resources permit, test post-ECLG questionnaires by administering them to monolingual individuals. Monolingual testing is a way to identify potential problems such as language errors, or cultural discordance and to find solutions before they result in patterns of non-response during actual data collection.[13]

Monolingual women may be identified by bilingual liaisons directly, through organizations working with the target populations (e.g., advisory committee), or through referrals from ECLG members. The bilingual liaisons should be comfortable with the study subject matter, since those who exhibit hesitance and discomfort may have greater difficulty working with monolingual women. To maximize the diversity of monolingual women identified, bilingual liaisons from each language group and country should be hired to work with monolingual women.[13]

The identified monolingual women should be fluent in one of the target languages but not in the source language. Recruited monolingual women should:

- reflect the target population as much as possible;
- speak one of the target languages;
- not be multi-lingual;
- have given birth in the past year;
- have lived in the host country for ≤ 5 years.

For monolingual testing, approximately 3-4 women per language should be included; however, identifying monolingual women who share key characteristics with the target population is more important than the actual number.[14] Introductions should be made at the beginning of the session including clarification of the roles of the research team and bilingual liaisons. The researcher should interact with the women throughout the meeting either with the help of the liaison or directly if the researcher is bilingual. Ensuring on-going communication between

the researchers and the monolingual women through the bilingual liaisons throughout the process is a way to enhance collaboration and quality of collected data.[13]

The bilingual liaison should first administer the MFMCQ in the target language. The researcher and bilingual liaisons should then ask the monolingual women to identify any administrative issues, and to ask them to suggest changes for any issues identified. Grammar, cultural relevance, and ease of understanding of the questions should be considered. All comments should be noted. The researcher and bilingual liaisons should verify any comments made that require clarification directly with the women. Babysitting services, a meal, and compensation should be offered to all monolingual women.

Any suggested changes to the source language version should be noted using track changes, “comments”, or handwritten on a hard copy of the source language version and a rationale provided. Refer these suggested changes back to ROAM.

All changes made to the target version between the ECLGs and monolingual testing should be highlighted. The footer of each page of the new target language version of the MFMCQ after monolingual testing should include “*MFMCQ- post monolingual testing of (*insert: *target language) – (*insert: *date)*”. The date ensures that the most recent version is always used.

8. Pilot-testing of the final target language version questionnaire

If the questionnaire was not administered to ECLG members prior to the ECLG meetings, pilot-test the back-translated target version of the MFMCQ (preferably over the phone) with a sample of the target study population. Select a sample of 5-6 individuals per language group for the pilot test (this number is adequate when the aim of the pilot is to assess clarity, wording, and acceptability of the study tool). Pilot-testing will likely require approval by an institutional research ethics review board.

Prior to administering the questionnaire:

- Communicate the aim of the study, and that the information will remain confidential
- Obtain consent

Post-completion, note:

- Duration of time to completion
- Systematically skipped questions (i.e., item non-response rates)
- Items with little or no variation in responses
- Respondents’ comments on the MFMCQ as a measurement tool

Any suggested changes to the source language version should be noted using track changes, “comments”, or handwritten on a hard copy of the source language version and a rationale provided. Refer these suggested changes back to ROAM.

All changes made to the target version between monolingual testing and pilot testing should be highlighted. The footer of each page of the new target language version of the MFMCQ after pilot testing should include “*MFMCQ- post pilot testing of (*insert: *target language) – (*insert: *date)*”. The date ensures that the most recent version is always used.

9. Provision of the final target language questionnaire to ROAM

Complete the **‘MFMCQ TRANSLATION AND VALIDATION COMPANION CHECKLIST’** and return it, together with the **WORD AND PDF VERSIONS OF THE FINAL NEW TARGET LANGUAGE MFMCQ** to ROAM at crothroam@gmail.com and anita.gagnon@mcgill.ca.

MFMCQ Translation & Cultural Validation Companion Checklist

Target Language: ____________________

Source Language: ____________________ Version Date: ____________________

Location (city, country) of validation work: ____________________

Date of completion of checklist: ____________________

Name of individual(s) completing checklist: ____________________

Contact information: ____________________

Instructions: Check the box for each item completed, and include relevant comments. Provide a justification for any deviations from optimal protocol components (**in bold**)

Step Description	Item (bolded are minimal requirements)	☑	Comments
1. Creation of and consultation with an advisory committee	Committee is diverse (i.e., researchers, health care professionals, and representatives from non-government organizations and government agencies)	☐	
	List of committee members has up-to-date contact information	☐	
	Source language questionnaire reviewed and approved by the committee	☐	
2. Translation & adaptation of the questionnaire from the source to the target language	**Source questionnaire is a culturally validated version**	☐	
	The translator's native language is the target language	☐	
	**Translator is fluent in both the source and target languages and familiar with associated cultures**	☐	
	Translator learned the source and target languages at different times & in different cultures *(Briefly describe)*	☐	
	**Translator is familiar with the purpose of the questionnaire and the intended respondents**	☐	
	**Translator is instructed to refer to Brislin's guidelines**	☐	
	**Translator is instructed to use wording understood by individuals with ≤ 5 years of formal education**	☐	
	**The footer of the new target language version reads: *"MFMCQ-original translation to (insert: target language) – (insert: date)"*.**	☐	
3. Assessment of the readability of the initially translated target language questionnaire	**Brislin's guidelines are consulted and incorporated as appropriate**	☐	
	**Sentences used are short and simple**	☐	
	**Any vague or general terms are replaced with specific terms**	☐	
	**No metaphors, idioms, or colloquialisms**	☐	
	**Readability level ≤ 5 years of formal education**	☐	
	If a readability tool is used, it is language-appropriate	☐	
4. Back-translation of the questionnaire	**The back-translator has not read the source language version or other target language versions of the questionnaire**	☐	
	The back-translator's native language is the source language	☐	
	**Back-translator is fluent in both the target and source languages and familiar with associated cultures**	☐	

MFMCQ Translation & Cultural Validation Companion Checklist

Target Language: ______ Source Language: ______ Version Date: ______ Location (city, country) of validation work: ______ Date of completion of checklist: ______ Name of individual(s) completing checklist: ______ Contact information: ______	**Instructions:** Check the box for each item completed, and include relevant comments. Provide a justification for any deviations from optimal protocol components (**in bold**)

Step Description	Item (bolded are minimal requirements)	☑	Comments
	Back-translator has a different background (culture, religion, etc.) than the original translator<br>*(Briefly describe the backgrounds of the translator & back-translator)*	☐	
	***If back-translation is written*, the back-translator works independently and does not consult the translator who completed the original translation.**	☐	
	***If back-translation is oral*, the back-translator orally back-translates all questions from the target-language version to the researcher and original translator**	☐	
	**Researcher and both translators discuss all discrepancies between the versions**	☐	
	**An agreement is reached on the optimal wording for all discrepancies arising in the target-language version**	☐	
	**Any suggested changes to the source-language version are noted using track changes, "comments" or handwritten on a hard copy, and a rationale provided**	☐	
	**All changes made to the target-language version between translation and back-translation are highlighted**	☐	
	**The footer of the new target language version after back-translation reads: *"MFMCQ-post back- translation of (insert: target language) – (insert: date)".***	☐	
5. Discussion with ECLGs	Bilingual liaisons who are comfortable with the subject matter are hired from each target language community	☐	
	Study aim & sample questions are shared with bilingual liaisons	☐	
	**The 'Best fit form' is completed for each ECLG member**	☐	
	**Profiles of ECLG members reflect the study population and the mix of ethno-cultural communities speaking the target language**<br>*(Briefly describe members' backgrounds)*	☐	
	**Overall 3-4 members fluent in the target language are consulted in the ECLGs**<br>*[Briefly describe format (e.g., group vs. individual)]*	☐	
	The questionnaire is administered over the phone to each member 1-2 days before the meeting; the duration of time to completion and any issues with administration are noted<br>*(Note the range of time to completion)*	☐	
	**The intended use of the translated questionnaire and purpose of the ECLG meeting is explained to members**	☐	
	**Questionnaire assessed for cultural appropriateness and acceptability (may use 'Cultural Appropriateness Scale')**	☐	
	**Questionnaire assessed for feasibility of completion**	☐	

MFMCQ Translation & Cultural Validation Companion Checklist

Target Language: ______<br>Source Language: ______ Version Date: ______<br>Location (city, country) of validation work: ______<br>Date of completion of checklist: ______<br>Name of individual(s) completing checklist: ______<br>Contact information: ______	**Instructions:** Check the box for each item completed, and include relevant comments. Provide a justification for any deviations from optimal protocol components (**in bold**)

Step Description	Item (bolded are minimal requirements)	☑	Comments
	**All answers to questions from "Instructions for conducting ECLG meetings" are recorded** *(Note questions not asked and why)*	☐	
	**All discrepancies between intended meanings and the group's understanding are reviewed, discussed, and suggestions are brought forward**	☐	
	**All issues raised are recorded with explanations**	☐	
	ECLG members are asked to provide contact information for women that may be appropriate for monolingual testing, and if they are willing to be contacted again	☐	
	**Babysitting services, a meal, and compensation is offered to all members and** time is allowed for members to interact	☐	
6. Incorporation of ECLG results	**All ECLG feedback is summarized and systematically reviewed by at least two researchers**	☐	
	Modifications are made to address issues raised by several ECLG members	☐	
	Modifications are made to address issues expected to compromise study participants' comfort and ability to respond	☐	
	**Any suggested changes to the source-language version are noted using track changes, "comments" or handwritten on a hard copy, and a rationale provided**	☐	
	**All changes made to the target-language version between back-translation and ELCGs are highlighted and a rationale provided for each**	☐	
	**The footer of the new target language version after ECLG input reads: *"MFMCQ-post ECLG of (insert: target language) – (insert: date)".***	☐	
7. Administration of translated questionnaires to monolingual women	**Target population is assessed for expected host country language capacity to determine if monolingual testing is necessary** *(If determined to be unnecessary, SKIP to Step 8: Pilot testing)*	☐	
	Researchers and bilingual liaisons discuss & clarify their roles prior to hiring monolingual women	☐	
	**Monolingual women are hired who reflect the study population and the geographical/cultural diversity within the target language** *(Briefly describe women's backgrounds)*	☐	
	**The monolingual women are fluent in the target language but not in the source language**	☐	
	Overall 3-4 women (per language) are included in the monolingual testing	☐	
	The bilingual liaison administers the MFMCQ in the target language to each of the monolingual women noting any issues with administration	☐	

MFMCQ Translation & Cultural Validation Companion Checklist

Target Language:	
Source Language:	Version Date:
Location (city, country) of validation work:	
Date of completion of checklist:	
Name of individual(s) completing checklist:	
Contact information:	

Instructions: Check the box for each item completed, and include relevant comments. Provide a justification for any deviations from optimal protocol components (**in bold**)

Step Description	Item (bolded are minimal requirements)	☑	Comments
	**The questionnaire is assessed for grammar, cultural relevance, and ease of understanding**	☐	
	**On-going communication is maintained between research staff and monolingual women through the bilingual liaisons**	☐	
	**All issues raised are recorded with explanations**	☐	
	**Babysitting services, a meal, and compensation is offered to all monolingual women**	☐	
	**Any suggested changes to the source-language version are noted using track changes, "comments" or handwritten on a hard copy, and a rationale provided**	☐	
	**All changes made to the target-language version between the ELCGs and monolingual testing are highlighted and a rationale provided for each**	☐	
	**The footer of the new target language version after monolingual testing reads: *"MFMCQ-post monolingual testing of (insert: target language) – (insert: date)".***	☐	
8. Pilot-testing of the final questionnaire with a sample of the target study population (*if necessary)	**It is determined if pilot-testing of the questionnaire is necessary (e.g. if questionnaire was not administered by phone to the ECLG members)** *(If determined to be unnecessary, SKIP to Step 9: Provision of final target language questionnaire to ROAM)*	☐	
	Institutional research ethics review board approval is obtained if required	☐	
	**The profile of respondents reflects the study population**	☐	
	The pilot-test sample includes 5-6 individuals per language group *(Note sample size and why it was deemed adequate)*	☐	
	**Consent is obtained prior to administering the questionnaire**	☐	
	**Duration of time to completion is recorded** *(Note the range of time to completion)*	☐	
	**Respondents' comments on the MFMCQ as a measurement tool, systematically skipped questions, and items with little or no variation are noted**	☐	
	**All issues raised are recorded with explanations**	☐	
	**Any suggested changes to the source-language version are noted using track changes, "comments" or handwritten on a hard copy, and a rationale provided**	☐	
	**All changes made to the target-language version between monolingual testing and pilot testing are highlighted and a rationale provided for each**	☐	

**MFMCQ Translation & Cultural Validation Companion Checklist**			
Target Language: ___ Source Language: ___ Version Date: ___ Location (city, country) of validation work: ___ Date of completion of checklist: ___ Name of individual(s) completing checklist: ___ Contact information: ___			**Instructions:** Check the box for each item completed, and include relevant comments. Provide a justification for any deviations from optimal protocol components (**in bold**)
**Step Description**	**Item (bolded are minimal requirements)**	☑	**Comments**
	**The footer of the new target language version after pilot testing reads*: "MFMCQ-post pilot testing of (insert: target language) – (insert: date)".***	☐	
9. Provision of the final target language questionnaire to ROAM	**Final target language MFMCQ is labeled *"MFMCQ – final (insert: target language) – (insert: date)"* in the document footer**	☐	
	**Send final new target language MFMCQ in Word and PDF formats to ROAM**	☐	
	**Send completed "MFMCQ Translation and Cultural Validation Companion Checklist" to ROAM**	☐	

REFERENCES

1. Geisinger KF. Cross-cultural normative assessment: Translation and adaptation issues influencing the normative interpretation of assessment instruments. Psychological assessment 1994;6:304.
2. Urquia ML, Gagnon AJ. Glossary: migration and health. Journal of Epidemiology and Community Health 2011;65:467-72.
3. Carlson ED. A Case Study in Translation Methodology Using the Health-Promotion Lifestyle Profile II. Public Health Nurs 2000;17:61-70.
4. Merry L, Gagnon A, Hemlin I, Clarke H, Hickey J. Cross-border movement and women's health: how to capture the data. International Journal for Equity in Health 2011;10:1-15.
5. Marin G, Marin BV. Research with Hispanic populations: Sage Publications, Inc; 1991.
6. Brislin RW. The wording and translation of research instruments. In: Lonner WJ, Berry JW, eds. Field methods in cross-cultural research Cross-cultural research and methodology series. Thousand Oaks: Sage; 1986:137-64.
7. Koller M, Aaronson NK, Blazeby J, et al. Translation procedures for standardised quality of life questionnaires: The European Organisation for Research and Treatment of Cancer (EORTC) approach. European Journal of Cancer 2007;43:1810-20.
8. Meade CD, Smith CF. Readability formulas: cautions and criteria. Patient Educ Couns 1991;17:153-8.
9. Paz SH, Liu H, Fongwa MN, Morales LS, Hays RD. Readability estimates for commonly used health-related quality of life surveys. Quality of Life Research 2009;18:889-900.
10. Contreras A, Garcia-Alonso R, Echenique M, Daye-Contreras F. The SOL formulas for converting SMOG readability scores between health education materials written in Spanish, English, and French. Journal of health communication 1999;4:21-9.
11. Ruppenthal L, Tuck J, Gagnon AJ. Enhancing Research With Migrant Women Through Focus Groups. Western Journal of Nursing Research 2005;27:735 -54.
12. Hertzog MA. Considerations in determining sample size for pilot studies. Research in Nursing and Health 2008;31:180-91.
13. Strohschein F, Merry L, Thomas J, Gagnon AJ. Strengthening Data Quality in Studies of Migrants not Fluent in Host Languages: A Canadian Example with Reproductive Health Questionnaires. . Research in Nursing and Health 2010.
14. Banville D, Desrosiers P, Genet-Volet Y. Translating questionnaires and inventories using a cross-cultural translation technique. Journal of Teaching in Physical Education 2000;19:374-87.
15. Rahman A, Iqbal Z, Waheed W, Hussain N. Translation and cultural adaptation of health questionnaires. JPMA - Journal of the Pakistan Medical Association 2003;53:142-7.
