# Supplementary material for: Development of the Migrant Friendly Maternity Care Questionnaire (MFMCQ) for migrants to Western societies: an international Delphi consensus process
Source: BMC Pregnancy Childbirth. 2014 Jun 10;14:200. doi: 10.1186/1471-2393-14-200 (PMC4088918; doi:10.1186/1471-2393-14-200)
Supplement: Additional file 4 — MFMCQ Spanish version_17Apr2014.pdf: the MFMCQ in Spanish. [file 1471-2393-14-200-S4.pdf]

Cuidados de Maternidad Amistosos a los Migrantes Cuestionario

Instrucciones para entrevistadores

1. Durante la entrevista, lee cada pregunta lentamente y claramente. Según las siguientes instrucciones, elige la respuesta más adecuada:
   - Para preguntas que piden *lee en voz alta y marca las casillas correspondientes* lee cada respuesta y deja la mujer que responda *sí* o *no* a cada opción.
   - Para preguntas que dicen *deja que la madre responde y marca las casillas correspondientes*, pregúntale sin darle opciones y después decide que opción conviene más a su respuesta. Si no logra pensar en alguna respuesta, lee 2-3 opciones para motivarla.

2. Sigue las instrucciones en cuanto a saltarse opciones
   Por ejemplo:
   P10 pregunta cuales servicios la madre hubiera preferido usar pero no lo hizo. Pregunta P11 "si no recibo el cuidado que querría para este embarazo, que barreras influyó esto" solo en caso de que la madre indica en P10 que había un servicio que querría usar.

3. Para cualquier pregunta que tiene que ver con un periodo de tiempo (por ejemplo P2, 6), incentívala a dar la mejor respuesta que pueda .

4. Si no estás seguro(a) de que casilla marcar (ej. complicaciones médicas o procedimientos) marca "Otro" y escribe una explicación

5. En caso de palabras que la mujer no entienda, usa las siguientes definiciones / explicaciones:
   - **Planificación familiar :** planificación en cuanto a tener hijos, y el uso de métodos anticonceptivos
   - **Infecciones trasmitidas sexualmente:** enfermedades o infecciones contraídas por relaciones sexuales
   - **Anestesia:** medicamentos que hagan que una persona no siente más o entumecen localmente o totalmente
   - **Unidad infantil de cuidados especiales:** la madre y el bebé fueron separados porque él bebé necesito estar bajo observación de los profesionales de la salud por alguna razón médica
   - **Aborto espontaneo:** embarazo perdido antes de 20 semanas de embarazo
   - **Embarazo interrumpido**: sacar el bebé antes de que se desarrollara; aborto
   - **Estatus de Inmigración (P93):** empieza el día de entrar en Canadá, y NO el día de recibir los documentos
   - **Centro de detención de inmigración:** maneras de inducir la respuesta: ¿Alguna vez fue encerrada por autoridades de inmigración? ¿Alguna vez fue encarcelada por razones de inmigración?
   - **Ingresos:** ingresos de todos los que viven en la casa (ejemplo, hermana, marido, madre) antes de los impuestos
   - **A cuántas personas sostiene:** la respuesta incluye la hermana, sus bebes, etc.
   - **Parto:** cuando las contracciones (los dolores) empezaron hasta el nacimiento del bebé.

NOTAS: (1) Se identificaron preguntas marcadas con * (n = 86) (durante un proceso de consenso Delphi con expertos internacionales de investigación en salud perinatal) como un conjunto mínimo de preguntas para su uso en comparaciones internacionales, (2) Las preguntas marcadas con M son sólo relevantes para las mujeres migrantes o identificadas como indicadores de migración recomendadas para capturar en el análisis de salud perinatal (Ver Gagnon AJ, Zimbeck M, Zeitlin J. Migration and Perinatal Health Surveillance: An International Delphi Survey. *European Journal of Obstetrics & Gynecology and Reproductive Biology*. 2010;149(1), 37-43).

El principio de la entrevista: El resumen del proyecto para las madres

Estoy trabajando con un grupo de investigación interesado en las experiencias de las mujeres inmigrantes en su nuevo país, durante el periodo de maternidad. Le voy a preguntar sobre sus experiencias durante el embarazo, el parto y el momento del nacimiento, el periodo después del nacimiento de su bebé, y la experiencia de maternidad en general. Después, le preguntaré sobre su historia obstétrica y su historia de inmigración. En todo momento durante la entrevista, puede pedirme que repita, clarifique o explique cualquier pregunta. Una vez más, quiero confirmarle que cualquier información que nos proporcione permanecerá confidencial. Si lo desea, puede retirarse del estudio en cualquier momento, como también puede elegir no responder a las preguntas que la incomoden.
Le pido el favor de informarme en cualquier momento si tiene alguna pregunta. Tiene alguna pregunta antes de que empecemos?
Entonces, comencemos!

MFMCQ Spanish Version		ID del estudio:	
HORA DE INICIO:		Nombre del entrevistador:	
HORA DE CONCLUSIÓN:		Fecha de la entrevista:	

1. ***M ¿En qué país nació? ______________**

---

2. ***M ¿Cuántos años hace que vive en este país?**
(El tiempo total en que la madre vivió en el país; en estatus de migración, las mujeres suelen ir y volver antes de mudarse definitivamente a un cierto país)

_____(días) ______(semanas) ______(meses) ______(años)

Esta primera serie de preguntas es sobre SU EMBARAZO MÁS RECIENTE en este país. Esta sección tiene 14 preguntas

---

3. **M ¿Llegó a este país embarazada con su último bebé?**

- ☐ Sí, cuántas semanas de embarazo tenía usted? ____________
- ☐ No
- ☐ No sabe

---

4. ***¿Recibió cuidados de un profesional de la salud para este embarazo (ej. un doctor, una enfermera, o una partera)?**

- ☐ Sí, ____________________(en qué países)
- ☐ No *(Pasa a la P8)*

---

5. ***¿Quién le atendió durante su embarazo en este país?**
(Deja que la madre responda y marca las casillas correspondientes; lee las opciones para motivarla si lo es necesario)

- ☐ Médico de cabecera/ médico general
- ☐ Obstetra, ginecólogo, OBGYN
- ☐ Partera
- ☐ Enfermera calificada para trabajar de doctor/ enfermera
- ☐ Otro (*por favor especifique*): _________
- ☐ N/A

---

6. ***¿Cuántas semanas de embarazo tenía cuando recibió cuidados por este embarazo por la primera vez?____________ (semanas); en este país?__________ (semanas)**
(Sin contar la visita destinada para hacer un test de embarazo)

- ☐ N/A (non he recibido cuidados por este embarazo)

---

7. ***¿Cuántas visitas tuvo con el doctor, la enfermera, o la partera durante este embarazo? _________**

- ☐ N/A (no he recibido cuidados por este embarazo)

---

8. ***¿Tuvo alguna complicación médica durante este embarazo?**

- ☐ Si ***(Deja que la madre responda y marca las casillas correspondientes)***
  - ☐ Anemia
  - ☐ Alta tensión arterial
  - ☐ Preeclampsia (hipertensión gestacional)
  - ☐ Parto antes de termino
  - ☐ Trombosis venosa profunda (TVP)
  - ☐ Diabetes gestacional
  - ☐ Placenta previa
  - ☐ Desprendimiento prematuro de placenta
  - ☐ Infección urinaria
  - ☐ Dolor de espalda intenso
  - ☐ Ruptura prematura de membrana
  - ☐ Depresión
  - ☐ Otro *(por favor especifique):* __________________________(complicaciones fetales incluidas)
- ☐ No, no tuvo usted ninguna complicación médica durante este embarazo

9. Durante este embarazo, ¿cuál de los siguientes servicios utilizó?

	Sí	*No*
Clases prenatales /clases de preparación al parto	☐	☐
Cita con un profesional de la salud	☐	☐
Banco de alimentos	☐	☐
Ayuda para encontrar alojamiento	☐	☐
Medicina tradicional/ rituales tradicionales	☐	☐
Servicios para la familia (ej. de guardería infantil, servicios de consultoría, cursos para padres)	☐	☐
Pruebas prenatales (ej. reconocimiento médico exhaustivo, pruebas de sangre, examen cervical, la prueba del Papanicolaou)	☐	☐
Pruebas para la detección de defectos congénitos (ej. Síndrome de Down)	☐	☐
Ecografía	☐	☐
Servicios de apoyo (ej. servicios para la salud mental)	☐	☐
Otro *(por favor especifique):* _________________	☐	☐

10. *Durante este embarazo, ¿quiso utilizar algunos de los siguientes servicios pero no pudo?
(Lee en voz alta y marca las casillas correspondientes)

☐ Clases prenatales /clases de preparación al parto
☐ Cita con un profesional de la salud
☐ Banco de alimentos
☐ Ayuda para encontrar alojamiento
☐ Medicina tradicional/ rituales tradicionales
☐ Servicios para la familia (ej. de guardería infantil, servicios de consultoría, cursos para padres)
☐ Pruebas prenatales (ej. reconocimiento médico exhaustivo, pruebas de sangre, examen cervical, la prueba del Papanicolaou)
☐ Pruebas para la detección de defectos congénitos (ej. Síndrome de Down)
☐ Ecografía
☐ Servicios de apoyo (ej. servicios para la salud mental)
☐ Otro *(por favor especifique):* _________________
☐ No *(Pasa a la P12)*

11. ***Si no recibió el cuidado que quiso durante este embarazo, ¿cuál eran los factores o las barreras que no se lo permitieron?**
(Deja que la madre responda y marca las casillas correspondientes)

- ☐ No se ofrecían estos servicios en su área
- ☐ Ya no había más lugar
- ☐ No sabía que ofrecían estos servicios
- ☐ No sabía que era elegible a estos servicios
- ☐ No era elegible a estos servicios
- ☐ No sabía dónde ofrecían estos servicios
- ☐ Temor a que afectara la solicitud de inmigración
- ☐ El profesional de la salud canceló la cita que tenía usted con él/ella
- ☐ Falta de servicios de guardería infantil
- ☐ Barrera lingüística
- ☐ No tenía transporte
- ☐ Razones económicas
- ☐ Tenía que trabajar
- ☐ No tenía tiempo
- ☐ Tenía que quedarse en casa
- ☐ Temor de las pruebas médicas o los reconocimientos médicos
- ☐ Recibe consejo y ayuda de su familia y amigos
- ☐ Los cuidados que recibió del sistema de salud no eran los que esperaba
- ☐ Tuvo dificultad para comprender cómo funciona "el sistema de salud" y/o problemas en la utilización del "sistema de salud"
- ☐ Se avergonzó
- ☐ Razones administrativas (ej. no tiene seguro médico)
- ☐ Otras *(por favor especifique):* _____________
- ☐ N/A

---

12. ***Durante este embarazo, ¿quién o cuál era su más importante fuente de información relativo al embarazo, al parto y al momento de nacimiento?**
(Deja que la madre responda y marca las casillas correspondientes)

- ☐ Embarazos anteriores
- ☐ Familia o amigos
- ☐ Líder espiritual
- ☐ Obstetra, ginecólogo, OBGYN
- ☐ Médico de cabecera/ médico general
- ☐ Partera
- ☐ Enfermera/ Enfermera calificada para trabajar de doctor
- ☐ Clases prenatales/ clases de preparación al parto
- ☐ Libros
- ☐ Televisión
- ☐ Internet
- ☐ Otro *(por favor especifique):* _____________

---

13. *** M Durante su embarazo, el profesional de la salud le dio información en su idioma?**

- ☐ Sí *(por favor especifique):* ________________
- ☐ No

---

14. ***Durante ESTE embarazo, y antes de su parto y el momento de nacimiento, ¿tuvo suficiente información sobre los siguientes asuntos?**

	Sí	*No*	*No sabe*
Cambios físicos durante el embarazo	☐	☐	☐
Cambios emocionales durante el embarazo	☐	☐	☐
Señales de advertencia del parto	☐	☐	☐
Medicamentos	☐	☐	☐
A qué atenerse durante el parto/ al momento de nacimiento	☐	☐	☐
Terapia del dolor sin medicamentos	☐	☐	☐
Pruebas médicas requeridas	☐	☐	☐
Nutrición durante el embarazo	☐	☐	☐
Su propia salud y recuperación después de dar a luz	☐	☐	☐
Cambios de humor que pueda tener	☐	☐	☐
Como atender a su bebe	☐	☐	☐
Como reconocer problemas con el salud y el desarrollo de su bebe	☐	☐	☐
Amamantamiento	☐	☐	☐
Alimentar con la formula	☐	☐	☐
A quien contactar si tiene preguntas sobre su salud o la de su bebe	☐	☐	☐
Planificación familiar/ control de la natalidad	☐	☐	☐
VIH y otras enfermedades trasmitidas sexualmente	☐	☐	☐

15. ***¿El profesional de la salud le preguntó sobre su plan para alimentar al bebé?**

☐ Sí
☐ No
☐ No sabe/ no se acuerda
☐ N/A (no tenía un profesional de la salud)

16. ***¿El profesional de la salud le preguntó si tenía alguna preferencia en cuanto a su cuidado, o si quería practicar alguna costumbre particular suya durante el embarazo?**

☐ Sí
☐ No
☐ N/A (no tenía un profesional de la salud)

La siguiente serie de preguntas es sobre EL PARTO Y EL MOMENTO DEL NACIMIENTO de su RECIEN NACIDO. Hay 16 preguntas en esta sección.

17. ***¿Cuántas semanas de embarazo tenía cuando dio a luz? __________ (semanas)**

☐ No sabe

18. ***¿A cuántos bebés dio a luz? _______________(ej. un solo niño, gemelos, etc.)**

---

19. ***¿Cuánto pesaba(n) su(s) bebé(s) al nacer?**

________(kg) ________(grams)/ _______(lbs) ________(oz)

________(kg) ________(grams)/ _______(lbs) ________(oz) ***(si dio a luz a múltiples)***

---

20. ***¿Dónde estuvo cuando dio a luz?**
(Lee en voz alta y marca la casilla correspondiente)

- ☐ En una sala de partos del hospital
- ☐ En una sala de operación del hospital
- ☐ En una sala de emergencia del hospital
- ☐ En una clínica
- ☐ En un centro de maternidad (fuera del hospital)
- ☐ En su casa
- ☐ Otro *(por favor especifique):* _____________

---

21. ***¿Qué tipo de profesional de la salud le atendió durante la mayor parte de <u>SU PARTO</u>?**
(Deja que la madre responda y marca la casilla correspondiente)

- ☐ Obstetra, ginecólogo, OBGYN
- ☐ Médico de cabecera/ médico general
- ☐ Partera
- ☐ Enfermera o enfermera calificada para trabajar de doctor
- ☐ Otro *(por favor especifique):*___________________
- ☐ Nadie
- ☐ Nadie, no hubo parto, cesárea planificada
- ☐ No sabe

---

22. ***¿Qué tipo de profesional de la salud le atendió mayormente <u>AL MOMENTO DE NACIMIENTO</u> de su bebé?**
(Deja que la madre responda y marca la casilla correspondiente)

- ☐ Obstetra, ginecólogo, OBGYN
- ☐ Médico de cabecera/ médico general
- ☐ Partera
- ☐ Enfermera o enfermera calificada para trabajar de doctor
- ☐ Otro *(por favor especifique):*_____________________
- ☐ Nadie
- ☐ No sabe

23. ***¿Pasó usted por alguno de los siguientes procedimientos durante el parto?**

	Sí	*No*
Inducción del parto (iniciar la contracciones)	☐	☐
Conducción del parto (hacer que las contracciones que usted ya tenía sean más fuertes y más frecuentes)	☐	☐
Uso de forceps (un instrumento metálico para sacar al bebé)	☐	☐
Uso de ventosa (instrumento de succión para sacar al bebé)	☐	☐
Operación cesárea	☐	☐
Episiotomía (incisión cerca de la abertura vaginal)	☐	☐
Epidural/peridural para aliviar el dolor durante el parto	☐	☐
Anestesia espinal para la cesárea	☐	☐
Anestesia general	☐	☐
Otro *(por favor especifique):* ____________	☐	☐

24. ***¿Hubo alguna complicación médica durante su parto y en el momento del nacimiento? (Como: desgarro perineal, ruptura uterina, infección, hemorragia post-parto o problemas con el bebé)**

☐ Sí *(por favor especifique):* ____

☐ No

Si el bebé nació por vía vaginal, pasa a la P26

25. ***Si su bebe nació por cesárea, ¿cuál fue la razón principal por la que realizó dicha operación?** ***(Deja que la madre responda y marca la casilla correspondiente)***

☐ Era planeada porque el doctor la sugirió por razones médicas
☐ Era planeada pero no sabía usted porque
☐ Era planeada porque usted la quiso, sin que haya razones médicas
☐ No era planeada, pero su parto duraba muchísimo
☐ No era planeada, pero el bebé estaba en peligro
☐ No era planeada, pero estaba usted en peligro
☐ No era planeada y no sabía usted porque ocurrió
☐ Otro *(por favor especifique):* ________________
☐ N/A (parto vaginal)

26. **Durante el parto, ¿pudo moverse o escoger posiciones cómodas sin restricción alguna?** ***(Lee en voz alta y marca la casilla correspondiente)***

☐ Sí, siempre
☐ Sí, algunas veces
☐ Sí, raramente
☐ No, por razones médicas
☐ No, no sabe por qué
☐ N/A, no hubo parto, cesárea planificada

27. Durante el parto, ¿los profesionales de la salud le preguntaron como quería tratar con el dolor?

- ☐ Sí
- ☐ No
- ☐ No sabe/ no se acuerda
- ☐ No, no hubo parto, cesárea planificada

---

28. Durante el parto, ¿estuvo satisfecha con la manera con la que los profesionales de la salud le ayudaron a tratar con el dolor?

- ☐ Sí
- ☐ No
- ☐ Algunas veces
- ☐ No, no hubo parto, cesárea planificada

---

29. Durante el parto, ¿pudo elegir a sus familiares y/o las personas de apoyo para estar con usted?

- ☐ Sí
- ☐ No
- ☐ Algunas veces
- ☐ No, no hubo parto, cesárea planificada

---

30. *¿Tenía algún compañero en el momento del nacimiento?
(Lee en voz alta y marca la casilla correspondiente)

- ☐ Sí, siempre
- ☐ Sí, algunas veces
- ☐ Sí, raramente
- ☐ No
- ☐ No sabe/ no se acuerda

---

31. *Si tuvo a alguien, ¿quién fue? (si tuvo a más de una persona, por favor mencione a todas)

___________________________(parentesco)
___________________________(parentesco)
___________________________(parentesco)

- ☐ N/A

---

32. *¿El profesional de la salud le preguntó si tenía alguna preferencia en cuanto a su cuidado, o si quería practicar alguna costumbre particular suya durante el parto o el nacimiento?

- ☐ Sí
- ☐ No
- ☐ No, porque les pregunte antes de que me preguntaran

Esta próxima serie de preguntas es sobre su periodo de POSTPARTO, el tiempo desde que su bebe nació. Hay 14 preguntas en esta sección.

33. *Después del parto, ¿su bebé necesitó recibir cuidados en una área separada de usted?
(Lee en voz alta y marca la casilla correspondiente)

☐ Sí, en una unidad neonatal de cuidados intensivos
☐ Sí, en una unidad especial para cuidar a los bebés
☐ Sí, en una unidad de cuidado neonatal
☐ Sí (pero usted no sabe/ no se acuerda)
☐ No
☐ No sabe/ no se acuerda

34. ¿Cuánto tiempo se quedó usted en el hospital o la clínica <u>después del nacimiento de su bebé</u>?

______(horas) _______(días) _______(semanas) _______(meses)

☐ N/A (nacimiento en casa)

35. ¿Siente que este tiempo fue muy corto/ muy largo/ o justo lo necesario?

☐ Muy corto
☐ Justo lo necesario
☐ Muy largo
☐ No sabría decir

36. ¿Los profesionales de la salud le preguntaron si tenía alguna preferencia alimentaria (por ejemplo: temperatura de la comida, comida preparada según sus creencias religiosas, comida vegetariana, u otro tipo de comida) durante su estancia en el hospital/ centro de maternidad?

☐ Sí
☐ No
☐ No sabe/ no se acuerda
☐ N/A (nacimiento en casa)

37. *¿Algunos de los profesionales de la salud le preguntaron si quería practicar alguna costumbre particular suya después de que naciese su bebé?

☐ Sí
☐ No
☐ No sabe/ no se acuerda

38. ¿En la primera hora después del nacimiento, le dieron su bebé para tenerlo piel a piel (tener su piel desnuda directamente en contacto con la suya)?

☐ Sí
☐ No. Si la respuesta es No, ¿por qué no?: ____

39. ***¿Cuándo fue que los profesionales de la salud le ayudaron o le ofrecieron ayuda para iniciar el amamantamiento?**
(Deja que la madre responda y marca la casilla correspondiente)

- ☐ Inmediatamente después del nacimiento
- ☐ No inmediatamente, pero mientras estaba en el lugar dónde dio a luz (ej. hospital, centro de maternidad, o en su casa)
- ☐ Más tarde, durante una visita /cita médica
- ☐ No le ayudaron y tampoco le ofrecieron ayuda
- ☐ No sabía/ no se acuerda
- ☐ No quiso amamantar a su bebe

---

40. ***¿Los profesionales de la salud le dieron información sobre los recursos de amamantamiento en su comunidad?**

- ☐ Sí
- ☐ No, pero usted no quiso la información *(Pasa a la P42)*
- ☐ No pero usted quiso la información *(Pasa a la P42)*
- ☐ No sabía/ no se acuerda

---

41. ***Si la respuesta es Sí, ¿pudo tener acceso a estos recursos de amamantamiento?**

- ☐ Sí
- ☐ No *(por favor especifique):* ____________
- ☐ N/A (no recibió información)

---

42. ***Después de dar a luz, ¿vio usted o su bebé a un profesional de la salud por razones relacionadas con este embarazo? (incluyendo citas de rutina)**

- ☐ Sí
- ☐ No *(Pasa a la P45)*
- ☐ No sabe/ no se acuerda

---

43. ***Si la respuesta es Sí, ¿por qué?_________________________**

- ☐ N/A (no vio a un profesional de la salud)

---

44. ***Si la respuesta es Sí, ¿a quién vio?**
(Deja que la madre responda y marca las casillas correspondientes.)

- ☐ Médico de cabecera/ médico general
- ☐ Médico de urgencias
- ☐ Obstetra, ginecólogo, OBGYN
- ☐ Partera
- ☐ Enfermera calificada para trabajar de doctor / enfermera
- ☐ Pediatra
- ☐ Otro *(por favor especifique):* ________________
- ☐ N/A (no vio a un profesional de la salud)

---

45. ***Desde que dio a luz, ¿quiso ver a un profesional de la salud para usted o su bebé pero no pudo?**

- ☐ Sí
- ☐ No *(Pasa a la P47)*

---

46. *Si no pudo ver a un profesional de la salud, ¿por qué no lo pudo?
(Deja que la madre responda y marca las casillas correspondientes)

- ☐ No se ofrecían estos servicios en su área
- ☐ Ya no había más lugar
- ☐ No sabía que ofrecían estos servicios
- ☐ No sabía que era elegible a estos servicios
- ☐ No era elegible a estos servicios
- ☐ No sabía dónde ofrecían estos servicios
- ☐ Temor a que afectara la solicitud de inmigración
- ☐ El profesional de la salud canceló la cita que tenía usted con él/ella
- ☐ Falta de servicios de guardería infantil
- ☐ Barrera lingüística
- ☐ No tenía transporte
- ☐ Por razones económicas
- ☐ Tenía que trabajar
- ☐ No tenía tiempo
- ☐ Tenía que quedarse en casa
- ☐ Temor de las pruebas médicas o los reconocimientos médicos
- ☐ Recibe consejo y ayuda de su familia y amigos
- ☐ Dificultades para comprender cómo funciona "el sistema de salud" y/o problemas en la utilización del "sistema de salud"
- ☐ Se avergonzó
- ☐ Razones administrativas (ej. no tiene seguro médico)
- ☐ Otras *(por favor especifique):* _____________
- ☐ No sabe/ no se acuerda

Esta serie de preguntas es sobre su EXPERIENCIA EN GENERAL CON EL SISTEMA DE SALUD DURANTE EL PERIDO DE MATERNIDAD, durante su embarazo más reciente. Hay 20 preguntas en esta sección.

47. Si usted lo piensa ahora, ¿había algún otro consejo/ apoyo/ información que hubiera querido recibir?

____

____

48. *En general, ¿cuándo veía a los profesionales de la salud, se sentía bienvenida por ellos/as?

a) Durante el embarazo

- ☐ Siempre
- ☐ Algunas veces
- ☐ Raramente
- ☐ Nunca

b) Durante el parto y al momento del nacimiento

- ☐ Siempre
- ☐ Algunas veces
- ☐ Raramente
- ☐ Nunca

c) Después del parto

- □ Siempre
- □ Algunas veces
- □ Raramente
- □ Nunca

---

49. *En general, ¿sintió que los profesionales de la salud fueron respetuosos?

a) Durante el embarazo

- □ Siempre
- □ Algunas veces
- □ Raramente
- □ Nunca

b) Durante el parto y al momento del nacimiento

- □ Siempre
- □ Algunas veces
- □ Raramente
- □ Nunca

c) Después del parto

- □ Siempre
- □ Algunas veces
- □ Raramente
- □ Nunca

---

50. *En general, ¿sintió que los profesionales de la salud le prestaban su ayuda?

a) Durante el embarazo

- □ Siempre
- □ Algunas veces
- □ Raramente
- □ Nunca

b) Durante el parto y al momento del nacimiento

- □ Siempre
- □ Algunas veces
- □ Raramente
- □ Nunca

c) Después del parto

- □ Siempre
- □ Algunas veces
- □ Raramente
- □ Nunca

51. ***En general, estuvo contenta con los cuidados que recibió**

a) Durante el embarazo

- ☐ Siempre
- ☐ Algunas veces
- ☐ Raramente
- ☐ Nunca

b) Durante el parto y al momento del nacimiento

- ☐ Siempre
- ☐ Algunas veces
- ☐ Raramente
- ☐ Nunca

c) Después del parto

- ☐ Siempre
- ☐ Algunas veces
- ☐ Raramente
- ☐ Nunca

---

52. ***Durante su embarazo, parto o al momento del nacimiento, ¿alguna vez los profesionales de la salud le pidieron hacer algo que usted no quería hacer?**

- ☐ Sí
- ☐ No
- ☐ No sabe/ no se acuerda

---

53. **Si la respuesta es Sí, ¿qué fue lo que le pidieron?**

____

- ☐ N/A

---

54. **¿Los profesionales de la salud le preguntaron sobre sus preferencias con respecto a tener un profesional de la salud que sea mujer o hombre?**

a) Durante el embarazo

- ☐ Siempre
- ☐ Algunas veces
- ☐ Raramente
- ☐ Nunca

Comentario:____________

b) Durante el parto y al momento del nacimiento

- ☐ Siempre
- ☐ Algunas veces
- ☐ Raramente
- ☐ Nunca

Comentario:____________

c) El primer día después del parto

- ☐ Siempre
- ☐ Algunas veces
- ☐ Raramente
- ☐ Nunca
- ☐ Comentario:_______________

---

55. *¿Comprendió la información que le dio el profesional de la salud?

a) Durante el embarazo

- ☐ Siempre
- ☐ Algunas veces
- ☐ Raramente
- ☐ Nunca

Comentario:_____________

b) Durante el parto y al momento del nacimiento

- ☐ Siempre
- ☐ Algunas veces
- ☐ Raramente
- ☐ Nunca

Comentario:_____________

c) El primer día después del parto

- ☐ Siempre
- ☐ Algunas veces
- ☐ Raramente
- ☐ Nunca

Comentario:_______________

---

56. * M ¿Hubiera comprendido mejor la información que le dio el profesional de la salud si hubiese sido comunicada en otro idioma?

- ☐ Sí, en qué idioma:_________________(ej. su lengua natal)
- ☐ No
- ☐ No sabe/ no se acuerda

---

57. * M ¿Los profesionales de la salud le ofrecieron servicios de interpretación?

a) Durante el embarazo

- ☐ Sí
- ☐ No
- ☐ N/A

b) Durante el parte y al momento del nacimiento

- ☐ Sí
- ☐ No
- ☐ N/A

c) El primer día después del nacimiento

- ☐ Sí
- ☐ No
- ☐ N/A

58. *** M ¿Con qué frecuencia tenía a alguien que hablaba su idioma y podía interpretar para usted?**

a) Durante el embarazo

- ☐ Siempre
- ☐ Algunas veces
- ☐ Raramente
- ☐ Nunca
- ☐ N/A

b) Durante el parto y al momento del nacimiento

- ☐ Siempre
- ☐ Algunas veces
- ☐ Raramente
- ☐ Nunca
- ☐ N/A

c) El primer día después del nacimiento

- ☐ Siempre
- ☐ Algunas veces
- ☐ Raramente
- ☐ Nunca
- ☐ N/A

---

59. *** M Si tenía a alguien quien interpretaba para usted, ¿quién fue?**
(Lee en voz alta y marca las casillas correspondientes)

a) Durante el embarazo

- ☐ Marido/pareja
- ☐ Otro miembro de la familia/ amigo
- ☐ Profesional de la salud
- ☐ Su hijo/a
- ☐ Un intérprete profesional
- ☐ Otro paciente o un amigo/ un miembro de la familia de otro paciente
- ☐ Otro *(por favor especifique):* ___________
- ☐ N/A

b) Durante el parto y al momento del nacimiento

- ☐ Marido/pareja
- ☐ Otro miembro de la familia/ amigo
- ☐ Profesional de la salud
- ☐ Su hijo/a
- ☐ Un intérprete profesional
- ☐ Otro paciente o un amigo/ un miembro de la familia de otro paciente
- ☐ Otro *(por favor especifique):* ___________
- ☐ N/A

c) En el primer día después del nacimiento

- ☐ Marido/pareja
- ☐ Otro miembro de la familia/ amigo
- ☐ Profesional de la salud
- ☐ Su hijo/a
- ☐ Un intérprete profesional
- ☐ Otro paciente o un amigo/ un miembro de la familia de otro paciente
- ☐ Otro *(por favor especifique):* ___________
- ☐ N/A

---

60. **[M]¿Estuvo satisfecha con aquellas interpretaciones?**

☐ Sí
☐ No
☐ No sabe/ no se acuerda
☐ N/A

---

61. ***Durante el parto, al momento del nacimiento o después del parto, ¿tuvo alguna preferencia con respecto a los cuidados o alguna costumbre o práctica en particular que quiso seguir pero no pudo porque el profesional de la salud lo prohibió/ o no arreglo que pueda usted hacerlo?**

☐ Sí
☐ No *(Pasa a la P64)*
☐ No sabe/ no se acuerda

---

62. **Si la respuesta es Sí, ¿cuáles fueron dichas preferencias?**

*i)*____________

*ii)*___________

*iii)*___________

☐ N/A

---

63. **Si la respuesta es Sí, ¿qué razón(es) le dio/dieron el/los profesional(es) de la salud para explicar porque no podía seguir estas preferencias?**

*i)*____________

*ii)*___________

*iii)*___________

☐ N/A

---

64. ***En su opinión, ¿hay alguna cosa que el profesional de la salud podría hacer de manera distinta o podría mejorar?**

a) Durante el embarazo

☐ Si *(Completa p65a)*
☐ No
☐ No sabe, no se acuerda

b) Durante el parte y al momento del nacimiento

☐ Si *(Completa p65b)*
☐ No
☐ No sabe, no se acuerda

---

c) Después del parto

- ☐ Si *(Completa p65c)*
- ☐ No
- ☐ No sabe, no se acuerda

---

65. Si la respuesta es Si, por favor describa como podía haber sido diferente o mejorado y por parte de quien:

a) Durante el embarazo
____

b) Durante el parto y al momento del nacimiento
____

c) Después del parto
____

---

66. *Por favor describa cualquier parte de su experiencia durante el embarazo, el parto, el momento del nacimiento o después del parto que la hizo sentir:

a) Particularmente contenta:
____

b) Particularmente descontenta:
____

Por favor dígame que tan seguido las siguientes 11 afirmaciones fueron ciertas durante su embarazo más reciente.

---

67. *Los profesionales de la salud me preguntaron si tenía alguna pregunta

- ☐ Siempre
- ☐ Algunas veces
- ☐ Raramente
- ☐ Nunca

---

68. Los profesionales de la salud estaban apresurados

- ☐ Siempre
- ☐ Algunas veces
- ☐ Raramente
- ☐ Nunca

69. ***Sentí que los profesionales de la salud atendieron seriamente mis preocupaciones**

a) Durante el embarazo

☐ Siempre
☐ Algunas veces
☐ Raramente
☐ Nunca
☐ N/A (no tuve cuidados prenatales)

b) Durante el parto y al momento del nacimiento

☐ Siempre
☐ Algunas veces
☐ Raramente
☐ Nunca
☐ N/A (no había un profesional de la salud presente conmigo)

c) Después del parto

☐ Siempre
☐ Algunas veces
☐ Raramente
☐ Nunca
☐ N/A (no había un profesional de la salud presente conmigo)

---

70. **Tuve que esperar mucho tiempo antes de recibir los cuidados**

a) Durante el embarazo

☐ Siempre
☐ Algunas veces
☐ Raramente
☐ No, nunca
☐ N/A (no tuve cuidados prenatales)

b) Durante el parto y al momento del nacimiento

☐ Siempre
☐ Algunas veces
☐ Raramente
☐ Nunca
☐ N/A (no había un profesional de la salud presente conmigo)

c) Después del parto

☐ Siempre
☐ Algunas veces
☐ Raramente
☐ Nunca
☐ N/A (no había un profesional de la salud presente conmigo)

71. ***Los profesionales de la salud me mantuvieron informada de lo que pasaba**

a) Durante el embarazo

- ☐ Siempre
- ☐ Algunas veces
- ☐ Si, raramente
- ☐ Nunca
- ☐ N/A (no tuve cuidados prenatales)

b) Durante el parto y al momento del nacimiento

- ☐ Siempre
- ☐ Algunas veces
- ☐ Raramente
- ☐ Nunca
- ☐ N/A (no había un profesional de la salud presente conmigo)

c) Después del parto

- ☐ Siempre
- ☐ Algunas veces
- ☐ Si, raramente
- ☐ Nunca
- ☐ N/A (no había un profesional de la salud presente conmigo)

---

72. ***Me sentí cómoda preguntando sobre cosas que no entendía**

a) Durante el embarazo

- ☐ Siempre
- ☐ Algunas veces
- ☐ Raramente
- ☐ Nunca
- ☐ N/A (no tuve cuidados prenatales)

b) Durante el parto y al momento del nacimiento

- ☐ Siempre
- ☐ Algunas veces
- ☐ Raramente
- ☐ No, nunca
- ☐ N/A (no había un profesional de la salud presente conmigo)

c) Después del parto

- ☐ Siempre
- ☐ Algunas veces
- ☐ Raramente
- ☐ Nunca
- ☐ N/A (no había un profesional de la salud presente conmigo)

73. ***Los profesionales de la salud tomaban decisiones a menudo sin tener en cuenta mis deseos**

a) Durante el embarazo

- ☐ Siempre
- ☐ Algunas veces
- ☐ Raramente
- ☐ Nunca
- ☐ N/A (no tuve cuidados prenatales)

b) Durante el parto y al momento del nacimiento

- ☐ Siempre
- ☐ Algunas veces
- ☐ Raramente
- ☐ Nunca
- ☐ N/A (no había un profesional de la salud presente conmigo)

c) Después del parto

- ☐ Siempre
- ☐ Algunas veces
- ☐ Raramente
- ☐ Nunca
- ☐ N/A (no había un profesional de la salud presente conmigo)

---

74. ***Los profesionales de la salud me daban muchos ánimos y seguridad**

a) Durante el embarazo

- ☐ Siempre
- ☐ Algunas veces
- ☐ Si, raramente
- ☐ No, nunca
- ☐ N/A (no tuve cuidados prenatales)

b) Durante el parto y al momento del nacimiento

- ☐ Siempre
- ☐ Algunas veces
- ☐ Si, raramente
- ☐ No, nunca
- ☐ N/A (no había un profesional de la salud presente conmigo)

c) Después del parto

- ☐ Siempre
- ☐ Algunas veces
- ☐ Si, raramente
- ☐ No, nunca
- ☐ N/A (no había un profesional de la salud presente conmigo)

75. *¿Los profesionales de la salud pasaron bastante tiempo con usted para darle explicaciones?

a) Durante el embarazo

☐ Siempre
☐ Algunas veces
☐ Si, raramente
☐ Nunca
☐ N/A (no tuve cuidados prenatales)

b) Durante el parto y al momento del nacimiento

☐ Siempre
☐ Algunas veces
☐ Raramente
☐ Nunca
☐ N/A (no había un profesional de la salud presente conmigo)

c) Después del parto

☐ Siempre
☐ Algunas veces
☐ Raramente
☐ Nunca
☐ N/A (no había un profesional de la salud presente conmigo)

---

76. *En general, ¿sintió usted que los profesionales de la salud le trataron de una manera diferente a las demás personas (ej: debido a su idioma o acento, cultura, su raza o color, su religión, su estatus migratorio, o su seguro médico)?

☐ Siempre *(Por favor especifique en P77)*
☐ Algunas veces *(Por favor especifique en P77)*
☐ Raramente *(Por favor especifique en P77)*
☐ Nunca *(Pasa a la P78)*

---

77. *Si la respuesta es Sí, ¿por qué razón(es) cree que fue tratada así?
(*Deja que la madre responda y marca las casillas correspondientes)*

☐ Idioma o acento
☐ Cultura
☐ Raza/ procedencia étnica
☐ Color de piel
☐ Religión
☐ Estatus migratorio
☐ Seguro médico
☐ Otra razón *(Por favor especifique):* _______________
☐ N/A

La siguiente serie de preguntas es sobre su HISTORIA OBSTÉTRICA. Hay 8 preguntas en esta sección.

78. ***¿Cuántos embarazos tuvo usted en total (incluyendo este embarazo)? ____________**

79. ***¿Cuántos embarazos acabaron en abortos espontáneos? ___________________**

- ☐ **N/A**

Ten cuidado al preguntar esto en frente de otras personas

80. ***¿Cuántos embarazos acabaron en abortos provocados? ______________**

- ☐ **N/A**

81. ***¿Cuántos embarazos acabaron en parto de feto muerto (el bebé falleció antes de nacer)? __________**

- ☐ **N/A**

82. ***¿Cuántos de sus bebés nacieron antes de 37 semanas cumplidas? __________**

- ☐ **N/A**

83. ***¿Cuántos de sus bebés nacieron después de 37 semanas cumplidas? ____________**

- ☐ **N/A**

84. ***¿Tuvo usted alguna complicación médica en sus embarazos pasados?**

- ☐ Sí
- ☐ No *(Pasa a la P86)*
- ☐ N/A *(Pasa a la P86)*

85. ***Si tuvo alguna complicación médica, ¿cuál eran?**
(*Deja que la madre responda y marca las casillas correspondientes*)

- ☐ Cesárea
- ☐ Anemia
- ☐ Alta tensión arterial
- ☐ Pre eclampsia (hipertensión gestacional)
- ☐ Parto antes de termino
- ☐ Trombosis venosa profunda
- ☐ Diabetes gestacional
- ☐ Placenta previa
- ☐ Desprendimiento prematuro de placenta
- ☐ Infección urinaria
- ☐ Dolor de espalda intenso
- ☐ Ruptura prematura de membrana
- ☐ Depresión
- ☐ Otro *(por favor especifique):* ___________________________
- ☐ No sabe

Esta última serie de preguntas es sobre USTED Y SU FAMILIA. Hay 27 preguntas en esta sección.

86. ***¿Cuál es su estatus marital?**

- ☐ Casada
- ☐ Unión libre
- ☐ Viuda
- ☐ Separada
- ☐ Divorciada
- ☐ Soltera

87. ***¿Con quién vive usted?**

	Sí	*No*
Esposo / Pareja (hombre)	☐	☐
Pareja (mujer)	☐	☐
Su madre/padre	☐	☐
Sus hermanos/hermanas	☐	☐
La madre/el padre de su pareja	☐	☐
Hermano(s)/hermana(s) de su pareja	☐	☐
Amigos(as)	☐	☐
Hijos(as) (otro que el recién nacido)	☐	☐
Otros *(por favor especifique):* ________________	☐	☐
Nadie, vivo sola con mi bebé	☐	☐
Nadie, vivo sola	☐	☐

88. ***¿Cuántos de sus hijos viven con usted (incluyendo el recién nacido)?** ____________

89. *** M ¿Cuántos de sus hijos nacieron en este país (incluyendo el recién nacido)?** ____________

90. ***¿Cuál es su fecha de nacimiento?** ______**(día)** __________________**(mes)** __________**(año)**

91. *** M ¿En qué país nació su madre?** ____________

92. *** M ¿En qué país nació su padre?** ____________

Las siguientes preguntas son para obtener información más detallada sobre su historial de migración. Estamos interesados en esta información porque queremos saber más sobre las experiencias de los inmigrantes a este país. Cualquier información que nos proporcione permanecerá confidencial; no se dará información a la oficina de migración. El responder a estas preguntas no afectará el estado de su demanda si ha aplicado por el estado de refugiado, residencia permanente o ciudadano.

93. *** M ¿Cuál es su estatus de inmigración actual?**
(Deja que la madre responda y marca la casilla correspondiente)

- ☐ Inmigrante (residente permanente)
- ☐ Refugiada
- ☐ Solicitante de estatus de refugiada o de asilo
- ☐ Trabajadora temporal/Asistencia doméstica
- ☐ Residente temporal
- ☐ Estudiante
- ☐ Visitante
- ☐ Sin estatus
- ☐ Sin documentación
- ☐ Ciudadana
- ☐ Otro *(por favor especifique):* _________________

94. **M ¿Cuánto tiempo hace que tiene usted este estatus?** _____**(días)** ______**(semanas)** ______**(meses)** ______**(años)**

95. **M ¿Cambió su estatus de inmigración desde que llegó?**

- ☐ Sí
- ☐ No *(Pasa a la P97)*

96. *** M Si la respuesta es Sí, ¿cuál era su estatus de inmigración anterior?**

- ☐ Inmigrante (residente permanente)
- ☐ Refugiada
- ☐ Solicitante de estatus de refugiada o de asilo
- ☐ Trabajadora temporal/Asistencia doméstica
- ☐ Residente temporal
- ☐ Estudiante
- ☐ Visitante
- ☐ Sin estatus
- ☐ Sin documentación
- ☐ Otro *(por favor especifique):* _________________
- ☐ N/A (no cambio de estatus)

---

97. **M ¿Alguna vez tuvo usted un estatus de refugiada?**

- ☐ Sí
- ☐ No
- ☐ No sabe/ no se acuerda

---

98. **M ¿Alguna vez ha estado en un centro de detención de inmigración?**

- ☐ Sí
- ☐ No *(Pasa a la P101)*

---

99. **M Si la respuesta es Sí, ¿durante cuánto tiempo? ______(días) ______(semanas) ______(meses) ______(años)**

- ☐ N/A

---

100. *** M Si la respuesta es Sí, ¿ha estado en un centro de detención de inmigración durante este embarazo?**

- ☐ Sí
- ☐ No
- ☐ N/A

---

101. ***¿Quién paga por sus servicios de salud?**

	Sí	*No*	*No sabe*
Un seguro médico público	☐	☐	☐
Un seguro médico privado	☐	☐	☐
Un seguro gubernamental especial para refugiados o solicitantes del estatus de asilo	☐	☐	☐
Usted paga por sus servicios de salud	☐	☐	☐

---

102. ***¿Cuál es su mayor título de estudios obtenido?**

- ☐ Escuela primaria
- ☐ Diploma de escuela secundaria
- ☐ Diploma terciario (ej. escuela de comercio, colegio, universidad)
- ☐ Diploma posgrado (Master, Doctorado)
- ☐ Ninguno

---

103. **M¿Legalmente, tiene usted derecho de trabajar en este país?**

- ☐ Sí
- ☐ No
- ☐ No sabe

---

104. ***¿Cuál fue su último trabajo salariado antes de que naciera el bebé?(ej. médico, profesor, operador entrada de datos, ayuda en asilos de ancianos, empleada de casa, cultivador de verduras y hortalizas, operador de máquinas de teñir textiles, ama de llaves de un hotel, centro de llamadas)**

*(por favor especifique):*___________________

- ☐ N/A (no trabajó)

---

105. ***¿Ha regresado al trabajo desde que nació su bebé?**

- ☐ Sí
- ☐ No

Explique por qué/ por qué no_____________________________ *(Si la respuesta es No, pasa a la P107)*

---

106. ***¿Si la respuesta es Sí, cuál es su trabajo actual?**

*(por favor especifique):*______________________

- ☐ N/A

---

107. ***¿Cómo califica usted el total de sus ingresos familiares (antes de impuestos)?**
(Inserta los valores que convienen a la situación del lugar y lee a voz alta)

- ☐ <_________ (Muy bajo)
- ☐ ___________ a ___________ (Bajo)
- ☐ ___________ a ___________ (Mediano)
- ☐ ___________ a ___________(Mediano-alto)
- ☐ >_________ (Alto)

---

108. ***¿A cuántas personas mantiene este ingreso (incluyendo al nuevo bebé)?** ______________

---

109. ***¿Qué idioma(s) habla con más frecuencia en casa?**

____

110. *** M ¿Qué tal habla el idioma de este país?**
(Aquí, inserta el idioma que conviene a la situación del lugar)

Language:

	Fluido	*Bien*	*Con dificultad*	*No, en lo absoluto*
Hablar	☐	☐	☐	☐
Leer	☐	☐	☐	☐
Escribir	☐	☐	☐	☐
Comprender	☐	☐	☐	☐

Haga la siguiente pregunta SÓLO si hay más de un idioma.

111. **M ¿Qué tal habla el idioma de este país?**
(Aquí, inserta el idioma que conviene a la situación del lugar)

Language:

	Fluido	*Bien*	*Con dificultad*	*No, en lo absoluto*
Hablar	☐	☐	☐	☐
Leer	☐	☐	☐	☐
Escribir	☐	☐	☐	☐
Comprender	☐	☐	☐	☐

112. ***Terminamos nuestra entrevista. ¿Hay alguna otra cosa que quisiera decir sobre los temas que hemos cubierto? ¿O alguna cosa más que quisiera añadir?**
